# The *Drosophila* BEAF insulator protein interacts with the polybromo subunit of the PBAP chromatin remodeling complex

**DOI:** 10.1093/g3journal/jkac223

**Published:** 2022-08-27

**Authors:** J Keller McKowen, Satya V S P Avva, Mukesh Maharjan, Fabiana M Duarte, Jacob M Tome, Julius Judd, Jamie L Wood, Sunday Negedu, Yunkai Dong, John T Lis, Craig M Hart

**Affiliations:** Department of Biological Sciences, Louisiana State University, Baton Rouge, LA 70803, USA; Department of Biological Sciences, Louisiana State University, Baton Rouge, LA 70803, USA; Department of Biological Sciences, Louisiana State University, Baton Rouge, LA 70803, USA; Department of Molecular Biology and Genetics, Cornell University, Ithaca, NY 14835, USA; Department of Molecular Biology and Genetics, Cornell University, Ithaca, NY 14835, USA; Department of Molecular Biology and Genetics, Cornell University, Ithaca, NY 14835, USA; Department of Biological Sciences, Louisiana State University, Baton Rouge, LA 70803, USA; Department of Biological Sciences, Louisiana State University, Baton Rouge, LA 70803, USA; Department of Biological Sciences, Louisiana State University, Baton Rouge, LA 70803, USA; Department of Molecular Biology and Genetics, Cornell University, Ithaca, NY 14835, USA; Department of Biological Sciences, Louisiana State University, Baton Rouge, LA 70803, USA

**Keywords:** BEAF, insulators, PBAP, chromatin remodeling complexes, gene regulation, *Drosophila*

## Abstract

The *Drosophila* Boundary Element-Associated Factor of 32 kDa (BEAF) binds in promoter regions of a few thousand mostly housekeeping genes. BEAF is implicated in both chromatin domain boundary activity and promoter function, although molecular mechanisms remain elusive. Here, we show that BEAF physically interacts with the polybromo subunit (Pbro) of PBAP, a SWI/SNF-class chromatin remodeling complex. BEAF also shows genetic interactions with Pbro and other PBAP subunits. We examine the effect of this interaction on gene expression and chromatin structure using precision run-on sequencing and micrococcal nuclease sequencing after RNAi-mediated knockdown in cultured S2 cells. Our results are consistent with the interaction playing a subtle role in gene activation. Fewer than 5% of BEAF-associated genes were significantly affected after BEAF knockdown. Most were downregulated, accompanied by fill-in of the promoter nucleosome-depleted region and a slight upstream shift of the +1 nucleosome. Pbro knockdown caused downregulation of several hundred genes and showed a correlation with BEAF knockdown but a better correlation with promoter-proximal GAGA factor binding. Micrococcal nuclease sequencing supports that BEAF binds near housekeeping gene promoters while Pbro is more important at regulated genes. Yet there is a similar general but slight reduction of promoter-proximal pausing by RNA polymerase II and increase in nucleosome-depleted region nucleosome occupancy after knockdown of either protein. We discuss the possibility of redundant factors keeping BEAF-associated promoters active and masking the role of interactions between BEAF and the Pbro subunit of PBAP in S2 cells. We identify Facilitates Chromatin Transcription (FACT) and Nucleosome Remodeling Factor (NURF) as candidate redundant factors.

## Introduction

The *Drosophila* Boundary Element-Associated Factor (BEAF) of 32 kDa was originally discovered based on binding to specific DNA sequences in the scs′ chromatin domain boundary, or insulator, element (Zhao *et al.* 1995). Insulators play roles in gene regulation, as shown by transgene assays. Communication between an enhancer and promoter is blocked by an insulator placed between them (Geyer and Corces 1992; Kellum and Schedl 1992) while bracketing a transgene with insulators protects it from chromosomal position effects (Kellum and Schedl 1991; Roseman *et al.* 1993). Evidence indicates insulators form the boundaries of topological domains, preventing regulatory communication and chromatin state spreading between domains (Chetverina *et al.* 2017; Chen and Lei 2019). Chromosomal rearrangements and mutations that affect insulators are associated with developmental abnormalities in flies and mammals due to gene misregulation (Vazquez and Schedl 2000; Guo *et al.* 2015; Lupiáñez *et al.* 2015; Franke *et al.* 2016; Arzate-Mejia *et al.* 2020; Ibrahim and Mundlos 2020).

BEAF is important for scs′ insulator activity (Gilbert *et al.* 2006; Roy, Gilbert, *et al.* 2007), and other tested genomic sequences containing BEAF binding sites also have insulator activity (Cuvier *et al.* 1998; Cuvier *et al.* 2002; Sultana *et al.* 2011; Schwartz *et al.* 2012). Yet genome-wide mapping found that BEAF binds in the promoter region of a few thousand mainly housekeeping genes (Bushey *et al.* 2009; Jiang *et al.* 2009; Nègre *et al.* 2010), suggesting a role in promoter function. In fact, Hi-C chromatin interaction mapping found that the boundaries between topologically associating domains (TADs) in *Drosophila* are usually occupied by housekeeping genes and the DNA-binding protein most frequently found at TAD boundaries is BEAF (Ulianov *et al.* 2016; Cubeñas-Potts *et al.* 2017; Hug *et al.* 2017). There are divergent promoters in scs′ (Glover *et al.* 1995) with separate BEAF binding sites near the transcription start site (TSS) of each (Zhao *et al.* 1995). Working with the *aurA* promoter region of scs′ we found that different but overlapping sequences, including the BEAF binding site, are key for promoter and insulator function (Maharjan *et al.* 2020). We also found that TSS-proximal BEAF binding can activate another housekeeping promoter, *RpS12*, but not the developmental *y* promoter (Dong *et al.* 2020). Thus, BEAF plays roles in both insulator and promoter activity, and these activities can be separated. Molecular mechanisms by which BEAF participates in either function remain unknown.

To gain insight into BEAF function, we used yeast 2-hybrid (Y2H) and coimmunoprecipitation coupled with tandem mass spectrometry to identify proteins that interact with BEAF. We previously reported that BEAF physically interacts with the transcription factor Serendipity-δ (Sry-δ) and promoter-proximal BEAF can facilitate activation of both the *RpS12* and *y* core promoters by distantly bound Sry-δ (Dong *et al.* 2020). Here, we provide evidence that BEAF physically interacts with the polybromo subunit (also called Bap180; hereafter referred to as Pbro for simplicity and to avoid confusion with proboscipedia) of the SWI/SNF-class PBAP chromatin remodeling complex. BEAF also shows genetic interactions with Pbro and other subunits unique to PBAP or shared with the related BAP complex (Mohrmann and Verrijzer 2005). Chromatin remodeling is important for gene regulation (Becker and Workman 2013), and PBAP has also been implicated in insulator function (Nakayama *et al.* 2012). Mammalian BAF (equivalent to *Drosophila* BAP) is enriched at active enhancers while PBAF (equivalent to *Drosophila* PBAP) is enriched at active promoters and 5′ ends of genes (Michel *et al.* 2018). PBAF has also been shown to help RNA polymerase II (Pol II) overcome the +1 nucleosome barrier (Nock *et al.* 2012). Mutations in human PBAF/BAF subunits are common in various cancers (Mittal and Roberts 2020), with PBRM1 (the human Pbro homolog) mutations being the second most common mutation in clear cell renal cell carcinoma (Varela *et al.* 2011).

Using precision run-on sequencing (PRO-seq) and micrococcal nuclease sequencing (MNase-seq), we report the effects of RNAi knockdown of BEAF or Pbro on gene expression and nucleosome organization. Our results suggest that there are redundant mechanisms to keep housekeeping genes active when BEAF is knocked down; and the role of PBAP is more important at regulated genes including those that are associated with the GAGA factor (GAF), as has been previously shown (Nakayama *et al.* 2012; Judd *et al.* 2021). We identify Facilitates Chromatin Transcription (FACT) (Tettey *et al.* 2019) and the ISWI-class chromatin remodeling complex Nucleosome Remodeling Factor (NURF) (Judd *et al.* 2021) as candidate redundant factors at BEAF-associated genes. Nevertheless, genes most dependent on BEAF for promoter proximal pausing by Pol II also require Pbro for maximal pausing, and broad small decreases in pausing and fill-in of active promoter nucleosome-depleted regions are consistent with an interaction between BEAF and Pbro fine-tuning gene expression at many promoters.

## Materials and methods

### Immunoprecipitation and tandem mass spectrometry

A P-element based transgenic fly line with an insulated *FLAG-BEAF-32B-EGFP* gene expressed from its endogenous promoter (Avva and Hart 2016) on the X chromosome was used to collect 4- to 20-h embryos. These flies also had the wild-type *BEAF* gene. Control embryos were collected from *y^1^ w^67c23^* flies. Nuclear extracts were prepared as previously described (Zhao *et al.* 1995) and immunoprecipitations were done using anti-FLAG M2 coupled to magnetic beads according to the manufacturer’s protocol (Sigma-Aldrich M8823). Two experimental and 3 control coimmunoprecipitation samples passed quality control (SDS-PAGE followed by Western blot for BEAF and silver stain for total protein) and were sent to the Thermo Fisher Scientific Center for Multiplexed Proteomics at Harvard Medical School for tandem mass spectrometry analysis. Differences between the experimental and control samples were evaluated for statistical significance by the Student’s *t*-test and corrected for multiple testing using the Benjamini–Hochberg correction. Results are in Supplementary Table 1.

### Y2H

Polybromo sequences were PCR amplified from a cDNA (Drosophila Genomics Resource Center FI03643) and cloned into the EcoRI site of the GAL4 activation domain (AD) plasmid pOAD. Cloning BEAF sequences into the GAL4 DNA-binding domain (DBD) plasmid pOBD2 was previously described (Avva and Hart 2016), except the long leucine zipper (LLZ) sequence which was PCR amplified and inserted into the pOBD2 EcoRI site. Note that there are 2 BEAF isoforms made from 1 gene, BEAF-32A and BEAF-32B (Hart *et al.* 1997). BEAF-32B has the dominant DNA-binding activity (Roy, Gilbert, *et al.* 2007; Jiang *et al.* 2009), so it was used for full-length BEAF. All subregions of BEAF tested are common to both isoforms. Y2H assays were conducted using standard methods as previously described (Avva and Hart 2016). Between 50 and 100 colonies containing both plasmids (growth on plates lacking tryptophan and leucine) for each plasmid combination were transferred onto plates to score for reporter gene expression (growth on medium also lacking adenine and histidine, with X-α-Gal to score α-galactosidase expression; all colonies that grew also showed α-galactosidase expression).

### Pull-down assays

Proteins were expressed in *Escherichia coli* strain BL21, pLysS by growth at 25°C for 24 h in autoinduction medium ZYM-5052 for preparation of protein extracts as previously described (Studier 2005; Dong *et al.* 2020). Extracts containing Myc-tagged Pbro or parts and FLAG-tagged BEAF-32B were mixed and pull downs were done using anti-FLAG M2 beads. SDS-PAGE was done loading 10% of input Myc-tagged protein and 25% pulled down Myc-tagged proteins with or without FLAG-tagged BEAF. Proteins were detected on Western blots using anti-Myc (Santa Cruz Biotechnology) or anti-BEAF (Zhao *et al.* 1995) antibodies as previously described (Dong *et al.* 2020).

### Polytene chromosome immunostaining

Polytene chromosomes from larval 3rd instar salivary glands were immunostained using standard methods (Roy, Gilbert, *et al.* 2007). Rabbit anti-Pbro (kindly provided by S. Hirose) (Nakayama *et al.* 2012) and monoclonal mouse anti-BEAF (Developmental Studies Hybridoma Bank) (Blanton *et al.* 2003) were used at 1:100 dilutions and Texas Red or FITC goat anti-rabbit or goat anti-mouse (Jackson ImmunoResearch) were used at 1:500 dilutions.

### Rough eye genetic interaction assay

Genetic interactions between BEAF and PBAP/BAP subunits were tested using a previously described assay (Roy, Tan, *et al.* 2007). The assay uses a transgene encoding a dominant-negative form of BEAF (BID, for BEAF self-Interaction Domain) driven by a GAL4-inducible *UAS* promoter. Driving expression of heterozygous *UAS-BID* by a heterozygous *ey-GAL4* driver (Bloomington *Drosophila* Stock Center BDSC 5535) results in a mild rough eye. Fly crosses are done to determine whether or not heterozygous mutations, *UAS-RNAi* transgenes or *UAS-DN* (dominant-negative) transgenes show genetic interactions that enhance the rough eye phenotype. The following mutations were tested: *Pbro[EY14080]* (BDSC 20789); *SAYP[G0381]* (BDSC 11996); *BAP55[EY15967]* (BDSC 21174); *Snr1[01319]* (BDSC 11529); and *Bap170[G5986]* (BDSC 28471). The following *UAS-RNAi* lines were tested: *Pbro*: Vienna *Drosophila* Resource Center 108618; *SAYP*: BDSC 32346; *brm*: BDSC 31712; *Bap111*: BDSC 26213 and BDSC 35242; *Bap60*: BDSC 32503; *Bap55*: BDSC 31708; *Bap170*: BDSC 26308; *mor*: BDSC 35630; and *osa*: BDSC 31266. The following *UAS-DN* lines were used: *brm*: BDSC 59046 and *mor*: BDSC 59074. Flies of the desired genotypes (including controls lacking *UAS-BID*) were collected, processed, and photographed using a JEOL JSM-6610LV scanning electron microscope at 10 kV under high vacuum, as previously described (Roy, Tan, *et al.* 2007).

### S2 cell tissue culture and RNAi treatment

*Drosophila* S2 cells were grown at 25°C in M3 + BPYE medium with 10% fetal bovine serum (FBS) and antibiotic/antimycotic (anti/anti; 100 U/ml penicillin, 0.1 mg/ml streptomycin, 250 ng/ml amphotericin B) from 5 × 10^5^ to 10^7^ cells/ml. Cells at 5 × 10^6^ cells/ml were diluted 5-fold with serum-free M3 + BPYE + anti/anti and 150 µg of dsRNA was added to 15 ml of cells in T150 flasks. After incubating 45 min at 25°C, 15 ml of the same medium supplemented with 20% FBS was added to the cells. After 2.5 days another 150 µg of dsRNA was added followed by 30 ml of M3 + BPYE, anti/anti, and 10% FBS, and the cells were split into 2 new flasks. After another 2.5 days, the cells were harvested for isolation of nuclei.

Synthesis of dsRNA used a dsDNA template with a T7 RNA polymerase promoter on both ends. The dsRNA was 668 bp for BEAF, 493 bp for Pbro, and 835 bp for LacZ. The DNA templates were generated by PCR using the following primers: BEAF forward (CTAATACGACTCACTATAGGGAGCAAGGCCAAGACGCTGAG); BEAF reverse (CTAATACGACTCACTATAGGGAGCGCTGATTTGCCCATTTAC); Pbro forward (CTAATACGACTCACTATAGGGAGCACTACTACGACATTATCAGGG); Pbro reverse (CTAATACGACTCACTATAGGGAGCTCTGTGCGGGACAACTTTC); control LacZ forward (GAATTAATACGACTCACTATAGGGAGAGATATCCTGCTGATGAAGC); and LacZ reverse (GAATTAATACGACTCACTATAGGGAGAGCAGGAGCTCGTTATCGC).

### Western blot analysis of RNAi knockdown efficiency

Western blots were done using standard methods. Dilutions of the LacZ-RNAi control (1.5, 5, and 15 µl at 1 × 10^5^ cells/µl) were used to estimate knockdown efficiencies of the BEAF-RNAi and Pbro-RNAi samples (15 µl each at 1 × 10^5^ cells/µl). Rabbit anti-Pbro antibody (Nakayama *et al.* 2012) was used at 1:1,000 dilution; Lis laboratory stocks of rabbit anti-BEAF and guinea pig anti-Chro and anti-TFIIS were used at 1:2,000, 1:2,000, and 1:3,000, respectively. Secondary antibodies (LI-COR Biosciences) were IRDye 800CW donkey anti-rabbit (1:15,000) and IRDye 680LT donkey anti-guinea pig (1:20,000), both at 1 mg/ml. Imaging was done using a LI-COR Odessy imaging system.

### PRO-seq library preparation

Nuclei were isolated and PRO-seq libraries were prepared as previously described using 10 PCR cycles after adaptor addition and reverse transcription (Kwak *et al.* 2013; Mahat *et al.* 2016).

### MNase-seq library preparation

Cells were treated with 1% formaldehyde for 2 min at room temperature, and cross-linking was quenched by adding glycine to 125 mM and placing the cells on ice. Nuclei were isolated as for PRO-seq (Kwak *et al.* 2013), except they were stored in MD buffer (10 mM Tris-HCl, pH 8.0, 250 mM sucrose, 1 mM CaCl_2_, 60 mM KCl, 15 mM NaCl, 1 mM DTT). MNase digestions were done in 140 µl MD buffer on nuclei from 1.5 × 10^7^ cells with 4,000 U MNase (NEB gel units) at room temperature for 30 min, resulting in around 90% mononucleosomes. Reactions were stopped (360 µl 142 mM NaHCO_3_, 1.42% SDS, 350 mM NaCl, 17.5 mM EDTA, 2.8 mM EGTA), DNA was isolated, and MNase-seq libraries were prepared as previously described (Wei *et al.* 2012). After 4 PCR cycles, DNA in the size range of 80–220 bp (not including adapter sequences) was isolated and sequenced.

### PRO-seq and MNase-seq library sequencing

Libraries were sequenced by the Cornell Biotechnology Resource Center on an Illumina NextSeq 500. The 6 bar-coded PRO-seq libraries (biological replicates of the 3 RNAi treatments) were pooled and 75 nucleotide reads were obtained. The 6 bar-coded MNase-seq libraries were pooled and 2 × 32 nucleotide paired-end reads were obtained.

### Data processing

PRO-seq (single-end) and MNase-seq (paired-end) Fastq files were processed using the FastX toolkit (hannonlab.cshl.edu/fastx_toolkit/index.html) to filter, clip the Illumina adapters (fastx_clipper), trim to 26-mers (fastx_trimmer), and align to the *Drosophila melanogaster* dm3 reference genome with up to 2 mismatches using Bowtie for PRO-seq (Langmead 2010) and Bowtie2 for MNase-seq (Langmead and Salzberg 2012). PRO-seq reads correspond to the sense strand of RNA, so were converted to their reverse complement before alignment. A summary of sequencing yields and the number of reads that uniquely mapped to the genome is given in Supplementary Table 2. Replicates were highly correlated (Supplementary Fig. 1) and combined for further analyses except for DESeq2. Sequence data were deposited at NCBI Gene Express Omnibus (GEO) under accession number GSE197584.

Using a list of 9,452 nonoverlapping genes (Core *et al.* 2012), custom scripts were used with PRO-seq data to count promoter proximal (50-bp window with the most reads from −50 to 150 relative to the annotated TSS) and gene body (from 200-bp downstream of the TSS to 200-bp upstream of the annotated gene end) Pol II sequence reads and to calculate pausing indexes (PIs) (github.com/McKowen-JK/BEAF_Pbro). Custom scripts were used with MNase-seq data to select 120–180-bp DNA fragments and calculate nucleosome centers (github.com/McKowen-JK/BEAF_Pbro). Nucleosome centers were used to create 50-bp pseudo-fragments before converting to bigWig. MACS2 (Feng *et al.* 2012) was used to call BEAF S2 cell ChIP-seq peaks using GSE52962 (Liang *et al.* 2014). Only peaks with over 25 reads were retained (3,036 peaks). DESeq2 (Love *et al.* 2014) was used to identify promoter region and gene body differentially expressed genes. Lists of differentially expressed genes, as FlyBase FBtr numbers (Gramates *et al.* 2017), were loaded into ChIP-Atlas Enrichment Analysis and searched against dm3 TF and others All cell types with a Threshold for significance of 100 and a distance range of 500 bp from the TSS to find factors enriched around these TSSs (Oki *et al.* 2018).

FACT PRO-seq data (SSRP and control eGFP RNAi; GSE129236) (Tettey *et al.* 2019) were processed as described above. Paired-end NURF PRO-seq data (NURF301 and control LacZ RNAi; GSE149339) (Judd *et al.* 2021) were processed using the authors pipeline to obtain deduplexed bigwig files for further analysis as described above.

## Results

### Characterization of the interaction between BEAF and polybromo

We previously reported detecting an interaction between BEAF-32B and the Pbro subunit of the PBAP chromatin remodeling complex when screening a Y2H cDNA library for interactions with BEAF (Dong *et al.* 2020). The catalytic subunit of PBAP is the SWI/SNF-class ATPase Brahma (Brm). There are 2 Brm-containing chromatin remodeling complexes in *Drosophila*, PBAP and BAP. Excluding actin, there are 6 subunits common to both. PBAP is distinguished by the presence of Pbro, Bap170 and SAYP, while BAP has the unique subunit Osa (Chalkley *et al.* 2008). We immunoaffinity purified FLAG-tagged BEAF-32B from embryonic nuclear protein extracts and identified copurifying proteins by proteomic tandem mass spectrometry (Supplementary Table 1). In addition to Pbro, 2 PBAP/BAP subunits were found with a false discovery rate (FDR) of less than 0.05, and 3 others had an FDR of less than 0.1 (Table 1). The other subunits, including the PBAP subunits SAYP and Bap170 and the BAP-specific subunit Osa, were present but at higher FDRs. Based on this we decided to characterize the interaction between BEAF and Pbro.

**Table 1.** PBAP/BAP coIP-MS results.

Protein	Synonym	Flybase ID	# of quantified peptides	Ratio (coIP/mock coIP)	Benjamini–Hochberg FDR
PBAP specific					
polybromo	Bap180	FBgn0039227	3	1.35	0.0383
SAYP	e(y)3	FBgn0087008	2	0.54	0.1498
Bap170		FBgn0042085	1	0.92	0.4402
PBAP and BAP					
Bap60		FBgn0025463	7	2.33	0.0158
Mor	BAP155	FBgn0002783	15	2.34	0.0383
Bap55	Arp4	FBgn0025716	5	1.38	0.0593
Brm		FBgn0000212	2	1.60	0.0617
Bap111	dalao	FBgn0030093	4	1.61	0.0622
Snr1	BAP45	FBgn0011715	2	0.85	0.3399
BAP specific					
Osa	eld	FBgn0261885	4	0.93	0.4156

**Fig. 1.**
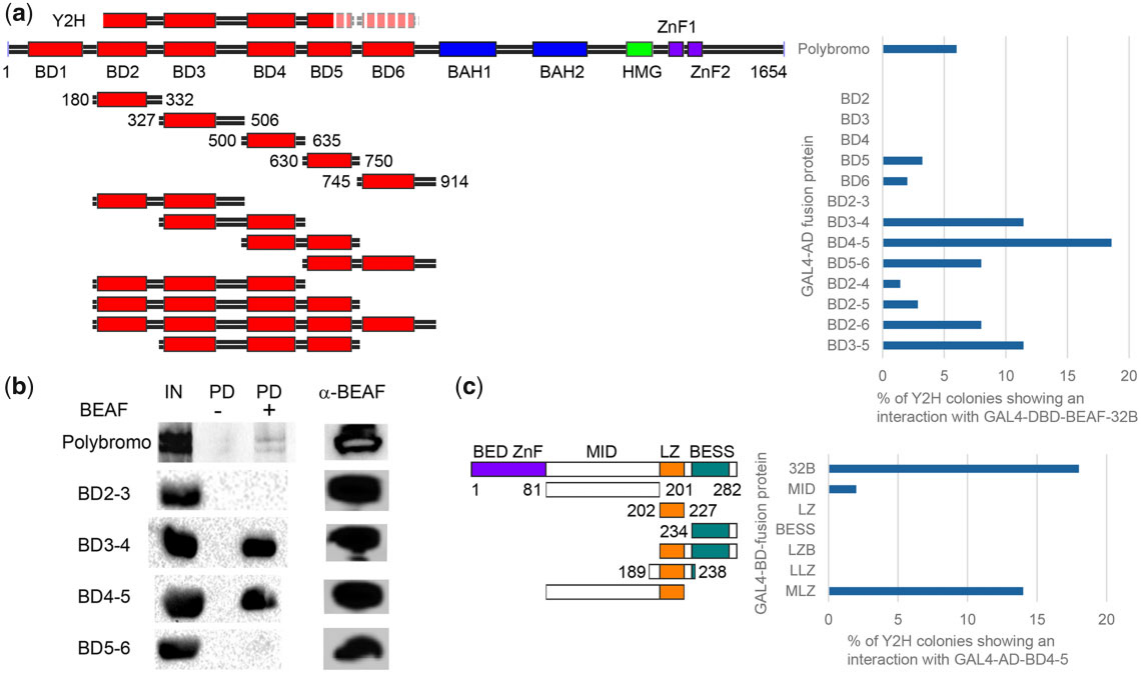
Mapping interactions between BEAF and Pbro using Y2H and pull-down assays. a) Schematic showing the domain organization of Pbro: 6 bromodomains (BD1 to BD6), 2 bromo-adjacent homology domains (BAH1, BAH2), a high mobility group domain (HMG), and 2 zinc-finger-like domains (ZnF1 and ZnF2). Above is shown the sequences present in the original Y2H GAL4 AD plasmid, indicating uncertainty in the carboxy endpoint (dashed light shading). Below are shown the GAL4 AD–bromodomain combinations tested for interaction with GAL4 DBD-BEAF-32B by Y2H. To the right are shown the % of Y2H colonies that expressed the reporter genes. The strongest interaction is with BD4–5, and at least 2 BDs were required to detect an interaction. b) Pull-down assays show that bacterially expressed Myc-tagged BD3–4 and BD4–5 interact with FLAG-BEAF-32B. IN: detection of Myc with 10% of input protein; PD −: detection of Myc with 25% of FLAG pull down in the absence of FLAG-BEAF-32B; PD +: detection of Myc with 25% of FLAG pull down in the presence of FLAG-BEAF-32B; α-BEAF: detection of 25% of pulled down FLAG-BEAF-32B. c) Left: Schematic of BEAF-32B and parts of BEAF tested for interaction with BD4–5 by Y2H. Right: % of Y2H colonies that expressed the reporter genes. LZB: leucine zipper plus BESS domain; MLZ: middle region plus leucine zipper. MLZ interacts with BD4–5.

We used Y2H to determine what part of Pbro interacts with BEAF (Fig. 1a). The library interaction plasmid had an insert of 1.4–2 kb and started just inside bromodomain (BD) 2, so it possibly ended in BD5 or extended just beyond BD6. We fused the GAL4 AD to the N-terminus of full-length Pbro and BD2–6 and tested for interaction with BEAF-32B with the GAL4 DBD fused at its N-terminus. In both cases, we found that not every colony containing both the AD and DBD plasmids grew on plates selecting for expression of the reporter genes (*HIS3*, *ADE2*, and *MEL1*). Only 5–10% of colonies grew, suggesting a weak or atypical interaction (such as 1 or both interacting surfaces requiring an unusual conformation or posttranslational modification). No single BD gave a convincing Y2H interaction, while of the adjacent BD combinations tested BD4–5 gave the strongest interaction with nearly 20% of colonies expressing the reporter genes. BD3–4 and BD5–6 pairs also showed interactions with BEAF-32B, while the presence of BD2 might interfere.

To follow up on these results, FLAG-tagged BEAF-32B and Myc-tagged adjacent BD pairs were expressed in *E. coli* and used in pulldown experiments using antibodies against FLAG (Fig. 1b). Full-length Pbro was also used, showing a weak pulldown that was absent in the control lacking FLAG-BEAF-32B. BD3–4 and BD4–5 showed strong pulldown. Taken together, the minimal interaction with BEAF includes 2 BDs. Although the results are ambiguous since BD3–4, BD4–5, and BD5–6 showed Y2H interactions, pairs with BD4 showed stronger interactions. Importantly, they support an interaction between BEAF and Pbro.

We next used Y2H to determine which part of BEAF interacts with Pbro BD4–5 (Fig. 1c). The structure of the middle region (MID) is unknown, although it is essential (Avva and Hart 2016) and we previously reported that the transcription factor Sry-δ interacts with it (Dong *et al.* 2020). The BESS domain interacts with itself to mediate interactions between BEAF subunits, and the presence of the putative leucine zipper (LZ) strengthens this interaction (Avva and Hart 2016). None of the regions tested alone or as the LZ-BESS combination interacted with BD4–5, while the MID-LZ combination did. To determine if the LZ alone can interact but the 25 amino acid LZ sequence does not fold properly, we lengthened LZ by adding around 10 amino acids to both sides (LLZ). This did not interact with BD4–5. Together, this suggests that the BEAF LZ plus some part of MID is responsible for the interaction with Pbro, preferentially with a pair of BDs including BD4. However, by Y2H this interaction appears weak and does not require BD4 since BD5–6 interacted.

**Fig. 2.**
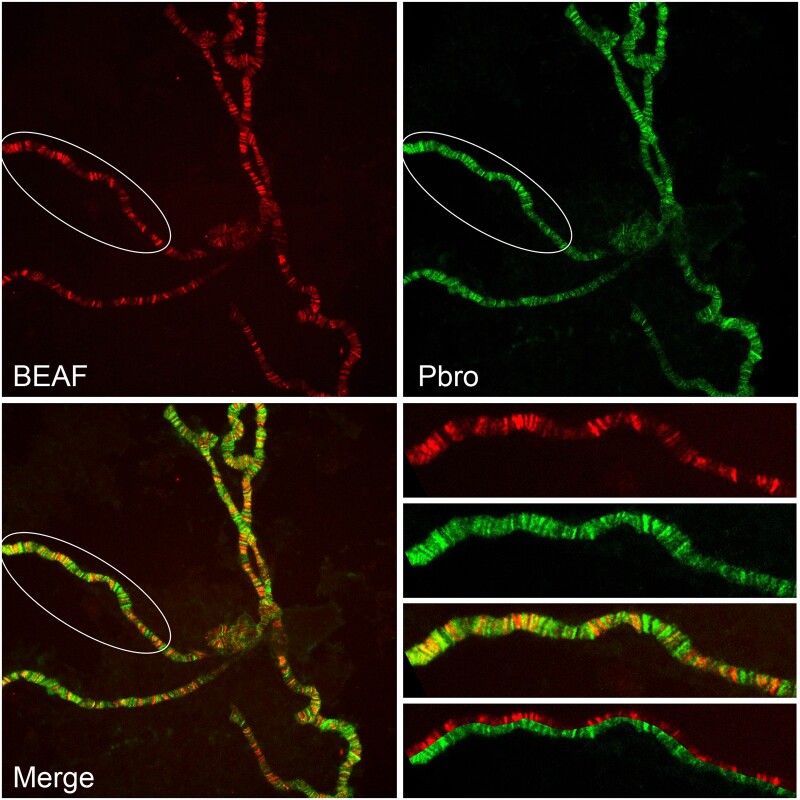
BEAF and Pbro partially overlap on polytene chromosomes. Immunolocalization of BEAF (upper left panel, red) and Pbro (upper right panel, green) on 3rd instar larval salivary gland polytene chromosomes. Partial overlap of these proteins is apparent in the overlay (lower left panel). The circled regions are enlarged in the lower right for a more detailed view (Top to bottom: BEAF, Pbro, Merged, Cutaway). See text for details.

To further examine the relationship between BEAF and Pbro, both were immunolocalized on polytene chromosomes (Fig. 2). As previously observed and expected for a sequence-specific DNA-binding protein, BEAF localized to hundreds of sharp bands. Pbro was more broadly distributed in a fuzzier pattern, consistent with being recruited to chromosomes by other proteins and possibly by histone modifications. Both proteins overlap, although their intensities often do not match. In particular, if BEAF recruits PBAP then Pbro would be expected to strongly colocalize with BEAF, but this is seldom the case. This is consistent with the Y2H indicating a weak interaction, such that BEAF bands generally coincide with diffuse, weak Pbro localization and most intense Pbro bands do not coincide with BEAF. We conclude that the extent of PBAP recruitment to BEAF sites depends on additional factors.

### BEAF and PBAP subunits show genetic interactions

**Fig. 3.**
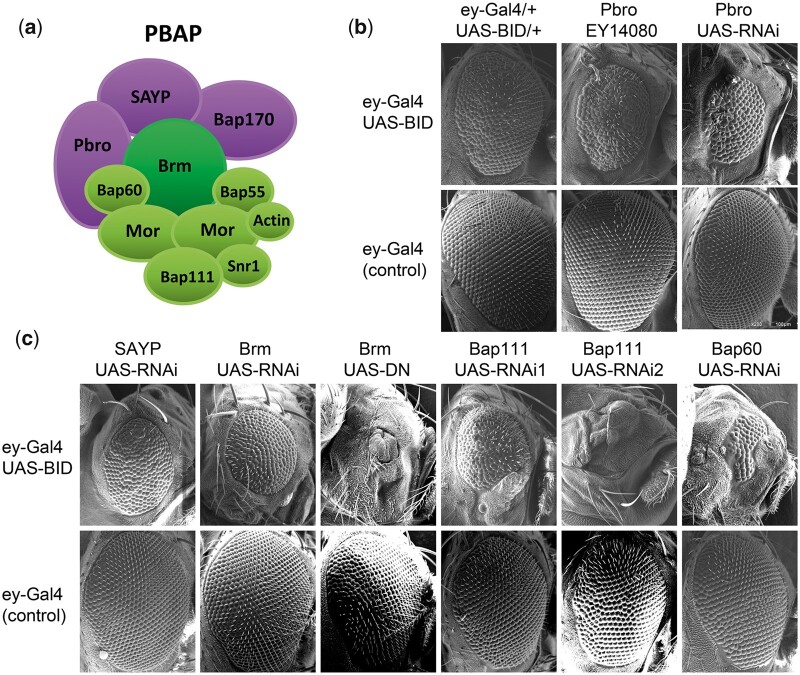
A rough eye assay reveals genetic interactions between BEAF and multiple PBAP subunits. a) Schematic of the PBAP complex. Subunits specific to PBAP are in purple while subunits also present in BAP are in green with the catalytic ATPase subunit in dark green. b) SEM images of the rough eye phenotype caused by heterozygous *UAS-BID* (encoding the dominant-negative BEAF self-Interaction Domain) driven by heterozygous *ey-GAL4*, and the enhancement of this phenotype by a heterozygous *Pbro* mutation or *UAS-RNAi* construct. Below are eyes from control flies lacking *UAS-BID*. c) SEM images of the enhanced rough eye phenotype caused by heterozygous *UAS-RNAi* transgenes against indicated PBAP subunits. Included is a *Brm UAS-Dominant-Negative* (DN) transgene. Control eyes are shown below.

A genetic interaction assay was used to test for a functional interaction between BEAF and PBAP subunits. Expression in eyes of a dominant-negative form of BEAF under GAL4 *UAS* control using an *ey-GAL4* driver results in a mild rough eye phenotype. When combined with *UAS-RNAi* transgenes or heterozygous mutations, the rough eye phenotype can be enhanced making this an effective screen for genetic interactions (Roy, Tan, *et al.* 2007). A cartoon of PBAP is shown in Fig. 3a indicating the 3 unique subunits and those shared with BAP. Both a mutant allele and a *UAS-RN*Ai line for Pbro enhanced the rough eye phenotype (Fig. 3b), so we tested at least 1 allele or *UAS-RNAi* line for the other subunits. Four other subunits, including the PBAP-specific SAYP, also showed genetic interactions with BEAF (Fig. 3c). SAYP is required for the stable association of Pbro and Bap170 into the PBAP complex (Chalkley *et al.* 2008). Other subunits did not show an interaction, including the BAP-specific protein Osa (Table 2 and Supplementary Fig. 2). Varying effects of RNAi knockdown of different PBAP/BAP subunits were expected since this was also reported in a study of mushroom body memory neurons (Chubak *et al.* 2019). The genetic interaction between BEAF and 5 PBAP/BAP subunits, including 2 PBAP-specific proteins and 2 different fly lines for 3 of the proteins, strongly argues for a functional connection between BEAF and PBAP.

**Table 2.** Summary of rough eye assay results.

Protein	Mutation	Rough eye enhancement		Fly stock
		+BID	−BID	
Pbro	UAS-RNAi	Yes	No	VDRC 108618
Pbro	[EY14080]	Yes	No	BDSC 20789
SAYP	UAS-RNAi	Yes	No	BDSC 32346
SAYP	[G0381]	No	No	BDSC 11996
Brm	UAS-RNAi	Yes	No	BDSC 31712
Brm	UAS-Dom Neg	Yes (strong)	Yes (weak)	BDSC 59046
Bap111	UAS-RNAi1	Yes	No	BDSC 26218
Bap111	UAS-RNAi2	Yes (strong)	Yes (weak)	BDSC 35242
Bap60	UAS-RNAi	Yes (strong)	No	BDSC 32503
Bap55	[EY15967]	No	No	BDSC 21174
Bap55	UAS-RNAi	No	No	BDSC 31708
Snr1	[01319]	No	No	BDSC 11529
Mor	UAS-RNAi	No	No	BDSC 35630
Mor	UAS-Dom Neg	No	No	BDSC 59074
Bap170	[G5986]	No	No	BDSC 28471
Bap170	UAS-RNAi	No	No	BDSC 26308
Osa	UAS-RNAi	No	No	BDSC 31266

### Transcriptional effects of BEAF and Pbro RNAi knockdown

**Fig. 4.**
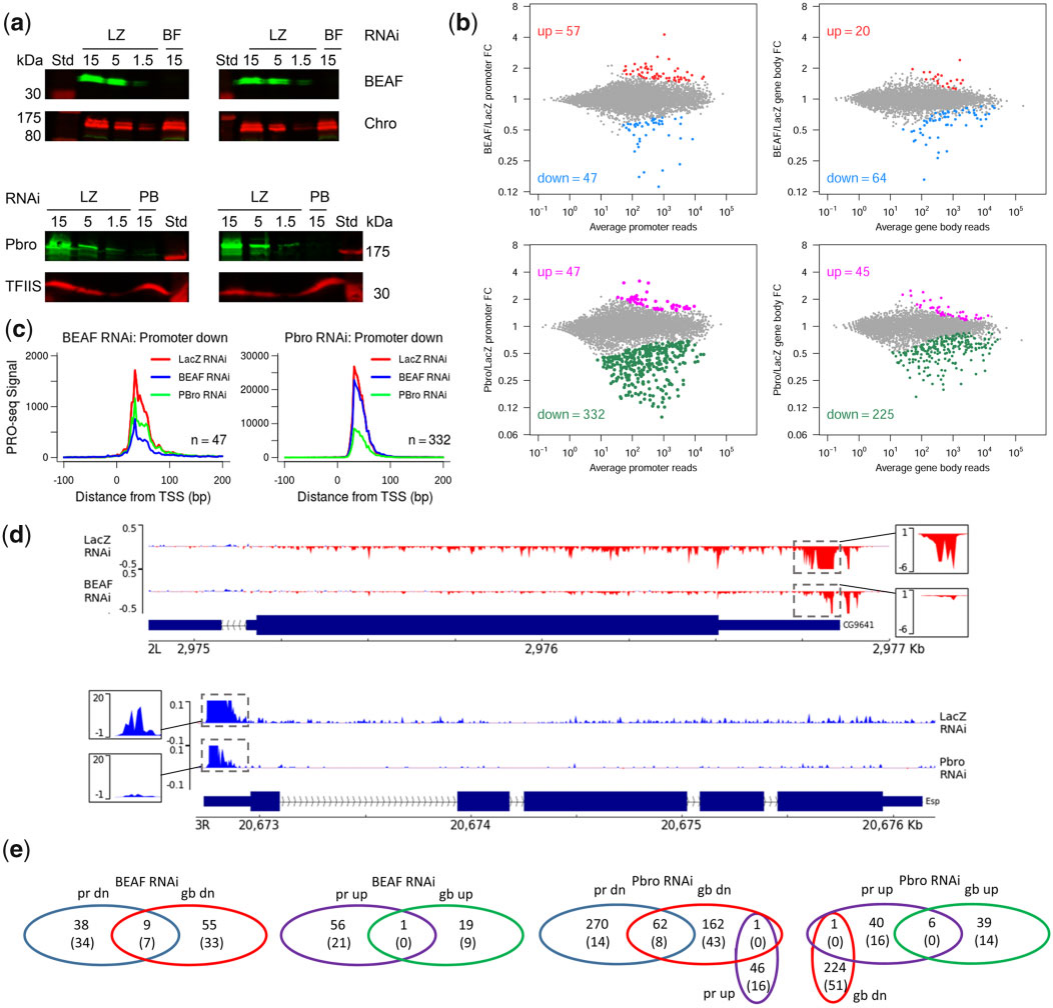
PRO-seq analysis of BEAF and Pbro RNAi knockdown effects on gene expression. a) Western blot of whole-cell extracts from LacZ (LZ), BEAF (BF), or Pbro (PB) RNAi-treated S2 cells using antibodies against BEAF, Chromator (loading control), Pbro, and TFIIS (loading control). Std: size standard, labeled in kDa. Fifteen is equivalent to 1.5 × 10^6^ cells. b) DESeq2 analysis of the effects of BEAF and Pbro, relative to control LacZ, RNAi on Pol II occupancy in the promoter proximal and gene body regions as determined by PRO-seq. Promoter proximal is the 50-bp window with the most reads in the region between −50 and +150 from the annotated TSS. Gene body is the region from 200-bp downstream of the TSS to 200-bp upstream of the annotated gene end. MA plots are shown with significantly upregulated and downregulated genes, defined as having an FDR of 0.05 or less, indicated by colored dots and numbers. FC: fold change. c) PRO-seq read density metaplots (in 3-bp bins) from −100 to +200 relative to the TSS for genes significantly downregulated in this region after BEAF knockdown (left graph) or Pbro knockdown (right graph). RNAi treatments, together with LacZ RNAi, are shown in each graph for the indicated number of genes (*n*). These metaplots show the largest change in gene expression compared to the other classes of differentially expressed genes shown in (b). Genes significantly downregulated by BEAF RNAi are also downregulated after Pbro RNAi. d) Genome browser views of a gene downregulated in both the promoter region and gene body after BEAF knockdown (top) or Pbro knockdown (bottom). Normalized PRO-seq tracks for cells treated with RNAi against the relevant gene and control LacZ are shown. Boxes adjacent to the promoter regions have the *y*-axis adjusted so promoter read peaks are on scale. e) Venn diagrams showing the overlaps in differentially expressed gene categories after BEAF or Pbro RNAi (pr: promoter region; gb: gene body; dn: downregulated; up: upregulated). Numbers in parentheses indicate the number of genes with a promoter-proximal BEAF ChIP-seq peak.

Insulators are implicated in gene regulation and BEAF usually binds near TSSs, often of housekeeping genes. Both the PBAP and BAP chromatin remodeling complexes play roles in gene expression. So we examined the effects of BEAF and Pbro RNAi-meditated knockdown on transcription in S2 cells using PRO-seq with LacZ RNAi as a control. Knockdown of BEAF was over 95%, and of Pbro was around 90% (Fig. 4a; full Western blots are shown in Supplementary Fig. 3). PRO-seq maps the location of transcriptionally engaged RNA Pol II with strand-specific nucleotide resolution, giving a snapshot of transcription at the time of nuclei isolation that is not affected by factors such as mRNA half-lives (Kwak *et al.* 2013). Biological replicates were highly correlated (Spearman’s coefficient of 0.98 for all 3 RNAi treatments in promoter and gene body regions; Supplementary Fig. 1).

Using a list of 9,452 nonoverlapping genes (Core *et al.* 2012), we counted promoter-proximal Pol II sequence reads and gene body Pol II sequence reads (see Fig. 4 legend). Defining transcriptionally active genes as having over 50 reads when combining promoter-proximal and gene body reads for both replicates (average for all genes was over 2,500 reads, median was over 475), 6,069 genes (64%) were active in the LacZ RNAi control (Table 3). DESeq2 (Love *et al.* 2014) was used with an FDR of 0.05 to identify genes whose reads were significantly changed in the promoter region or gene body (Supplementary Table 3).

**Table 3.** Active genes and Pol II promoter-proximal pausing data (*P*-value <0.01, Fisher’s exact test).

RNAi	Gene category	Genes	Active	Paused	Active and paused
LacZ	Total	9,452	6,069	5,194	5,040
	BEAF associated	2,535	2,488	2,282	2,278
BEAF	Total	9,452	6,081	4,991	4,854
	BEAF associated	2,535	2,486	2,193	2,190
Pbro	Total	9,452	5,973	4,866	4,747
	BEAF associated	2,535	2,486	2,201	2,197

First, we focused on the effects of BEAF knockdown. Using RNA-seq, 1 study reported that over 2,000 genes were differentially expressed after knockdown of BEAF in S2 cells, of which 57% had a BEAF ChIP-seq peak in their promoter regions (Lhoumaud *et al.* 2014). In contrast, another study found that BEAF knockdown in BG3 cells affected only 6 genes, all of which were downregulated (Schwartz *et al.* 2012). We found that 178 genes were differentially expressed (Fig. 4b), most of which were affected only in the promoter region (47 downregulated and 57 upregulated) or gene body (64 downregulated and 20 upregulated). Nine genes were downregulated and 1 gene was upregulated in both the promoter region and gene body. The clearest effect on gene expression was observed in metaplots of genes downregulated in the promoter region (Fig. 4c and Supplementary Fig. 4). An example of a gene downregulated in both the promoter region and gene body is shown in Fig. 4d.

Because BEAF usually binds near TSSs, we used S2 cell ChIP-seq data (Lhoumaud *et al.* 2014) to determine how many differentially expressed genes were BEAF associated. We found 3,036 BEAF peaks, and TSSs of 2,535 of the 9,452 genes we used were within 500 bp of the apex of one of these peaks. Using the above definition of active genes, 2,488 (98%) of these BEAF-associated genes were active. There was a better correlation between BEAF association and downregulation after BEAF knockdown (41 of 47 genes in the promoter region, 40 of 64 genes in the gene body, 7 of which were down in both regions) than for upregulation (21 of 57 genes in the promoter region, 8 of 20 genes in the gene body, none of which were up in both regions; Fig. 4e). This is consistent with promoter-proximal BEAF directly playing a greater role in gene activation (Emberly *et al.* 2008; Jiang *et al.* 2009; Dong *et al.* 2020; Maharjan *et al.* 2020). Our data also suggest that most BEAF-associated genes have redundant mechanisms for maintaining an active state and less than 5% were affected by BEAF knockdown at our significance threshold.

Knockdown of Pbro affected the expression of 580 genes at our significance threshold, over 80% of which were downregulated (Fig. 4b). Of these, 62 genes were downregulated in both the promoter region and the gene body; 6 were upregulated in both regions; and 1 was upregulated in the promoter region but downregulated in the gene body. Again, the clearest effect on gene expression was observed in metaplots of genes downregulated in the promoter region (Fig. 4c and Supplementary Fig. 4). An example of a gene downregulated in both the promoter region and gene body is shown in Fig. 4d. We did not observe a strong correlation between promoter-proximal BEAF and downregulation (22 of 332 genes in the promoter region, 51 of 225 genes in the gene body, 8 of which were down in both regions) or upregulation (16 of 47 genes in the promoter region, 15 of 45 genes in the gene body, none of which were up in both regions) (Fig. 4e). Also, there was not much overlap with differentially expressed genes after BEAF knockdown. The overlaps involved 38 genes and were a complicated mix of promoter region and gene body, down- and upregulation (Supplementary Table 4). The largest overlap category had 15 genes that were downregulated in the gene body after both BEAF and Pbro knockdown, of which 5 had promoter-proximal BEAF peaks. However, in support of a functional connection between BEAF and PBAP, genes that were significantly downregulated in the promoter region after BEAF knockdown were also downregulated after Pbro knockdown although to a lesser extent (Fig. 4c). As expected since genes affected by BEAF RNAi should at most be a subset of genes affected by PBAP RNAi, the converse is not true (Fig. 4c). Just as redundant mechanisms could compensate for the effects of BEAF knockdown at BEAF-associated promoters, the low overlap between BEAF-associated promoters and differentially expressed genes after Pbro knockdown could indicate that PBAP can be recruited to these promoters independently of BEAF. Another nonexclusive possibility is redundancy with other chromatin remodeling complexes at BEAF-associated promoters, such as BAP or the ISWI-based NURF complex.

Because of the poor correlation with BEAF, we used ChIP-Atlas to do an enrichment analysis for factors found within 500 bp of promoters of differentially expressed genes after Pbro knockdown (Oki *et al.* 2018). GAF was the most significantly differentially enriched factor for downregulated genes in the promoter region (log(*Q*-value) = −57.8) and gene body (log(*Q*-value) = −42.1) both separately and when combined (log(*Q*-value) = −87.0). This is consistent with reports of an association between PBAP and GAF (Nakayama *et al.* 2012; Judd *et al.* 2021). The results for upregulated genes were more variable, with higher *Q*-values because there were fewer genes. Different top differentially enriched factors were found when the analysis was done for genes differentially expressed in the promoter region (pipsqueak; log(*Q*-value) = −2.1), gene body (CP190; log(*Q*-value) = −5.2), or both gene lists combined (mod(mdg4); log(*Q*-value) = −5.0). However, GAF was still among the most significant factors.

### Effects of BEAF and Pbro RNAi knockdown on Pol II pausing

**Fig. 5.**
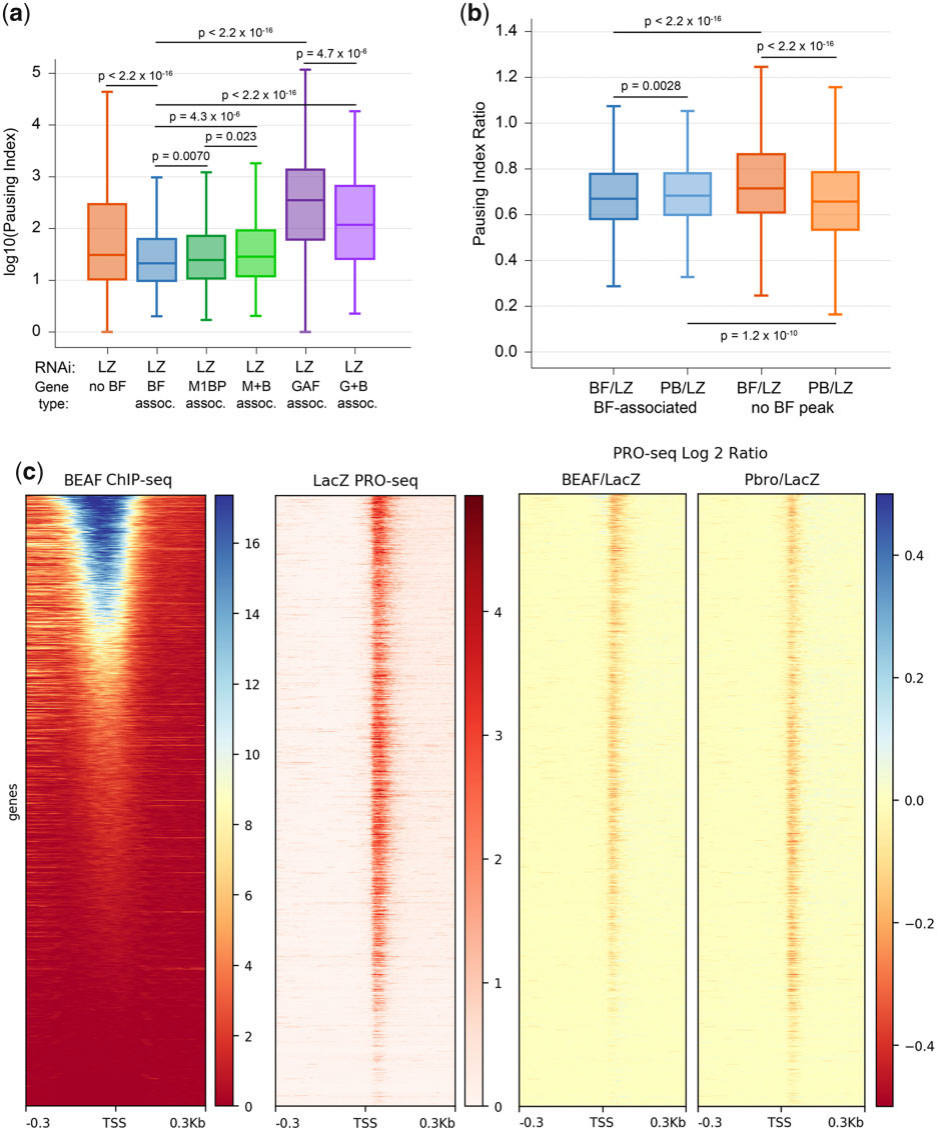
PRO-seq analysis of BEAF and Pbro RNAi knockdown effects on Pol II pausing. a) Box plot showing the range of log10 PIs of active, paused genes lacking a promoter-proximal BEAF peak (*n* = 2,762), or with a promoter-proximal peak for BEAF (*n* = 2,278), M1BP (*n* = 1,360), M1BP and BEAF (*n* = 767), GAF (*n* = 676), or GAF and BEAF (*n* = 213). Control LacZ RNAi data were used. Pausing of BEAF-associated genes is slightly lower than for M1BP-associated genes (MWU, *P*-value = 0.0070), and much lower than for GAF-associated genes and genes lacking BEAF (MWU, P-value <2.2 × 10^−16^ for both). Pausing at genes with both BEAF and M1BP was higher than for either alone (MWU, *P*-value = 4.3 × 10^−6^ for the comparison with BEAF-associated genes and 0.023 for comparison with M1BP-associated genes). Pausing at genes with both BEAF and GAF was higher than for BEAF (MWU, *P*-value <2.2 × 10^−16^) but lower than for genes associated with GAF (MWU, *P*-value = 4.7 × 10^−6^). Boxes depict the 25th through the 75th percentiles with the median indicated, and whiskers show the 10th through 90th percentiles. b) Box plot showing the PI fold-change for BEAF-associated genes and genes lacking a promoter-proximal BEAF peak, after RNAi treatment for BEAF or Pbro relative to the control LacZ. BEAF RNAi decreases pausing on BEAF-associated genes slightly more than does Pbro RNAi (MWU, *P*-value = 0.0028) and more strongly than BEAF RNAi on genes lacking a promoter-proximal BEAF peak (MWU, *P*-value <2.2 × 10^−16^). Pbro RNAi decreases pausing more than BEAF RNAi on genes lacking promoter-proximal BEAF (MWU, *P*-value <2.2 × 10^−16^) and more than Pbro RNAi on BEAF-associated genes (MWU, *P*-value = 1.2 × 10^−10^). c) Heatmaps showing the gene-by-gene effects of RNAi on pausing by Pol II in a 600-bp window centered on TSSs. Left: active genes arranged by BEAF ChIP-seq signal (*n* = 6,069). All heatmaps retain this gene order. Middle: heatmap of PRO-seq data from cells treated with the control LacZ RNAi, showing pausing by most active genes. Right: log2 heatmaps showing the fold-change in pausing of BEAF RNAi (left) or Pbro RNAi (right) relative to LacZ RNAi.

We next calculated the PI for each gene as another way to examine effects of BEAF and Pbro knockdown on gene expression (Supplementary Table 5). PI is the promoter-proximal Pol II density (promoter-proximal reads as defined above, divided by the number of mapped bases) divided by the gene body Pol II density (gene body reads as defined above, divided by the number of mapped bases) (Core *et al.* 2008). Focusing on the control LacZ RNAi treatment we found that most active genes were paused (∼83%, Fisher’s exact test, *P*-value <0.01), which is similar to previous reports (Fuda *et al.* 2015; Duarte *et al.* 2016). This increased to over 90% paused for active BEAF-associated genes (Table 3). The PI was significantly lower for active genes that were BEAF-associated relative to not BEAF-associated [Fig. 5a; Mann–Whitney *U*-test (MWU), *P*-value <2.2 × 10^−16^]. So pausing is a general feature of active genes, although BEAF-associated genes usually have weak pausing.

Like BEAF, Motif 1 Binding Protein (M1BP) is a sequence-specific DNA-binding protein found in the promoter region of many housekeeping genes (Li and Gilmour 2013). The sets of promoters bound by BEAF or M1BP have significant overlap with each other [from the list of 9,452 genes used here: as mentioned, 2,535 are BEAF-associated; 1,459 have an M1BP peak apex within 300 bp of a TSS (Li and Gilmour 2013); and 814 are associated with both]. GAF is associated with Pol II pausing (Lee *et al.* 2008; Li and Gilmour 2011; Fuda *et al.* 2015; Duarte *et al.* 2016), and it has been noted that BEAF and GAF sometimes colocalize with BEAF seeming to block interaction between upstream GAF and promoter-region Pol II (Fuda *et al.* 2015; Duarte *et al.* 2016). M1BP has been reported to orchestrate Pol II pausing through a mechanism distinct from that of GAF-mediated NELF recruitment (Li and Gilmour 2013). Because of the relationship between BEAF and M1BP and GAF, we compared the PI of BEAF-associated genes with the PI of M1BP and GAF-associated genes regardless of overlap between genes in different categories (Fig. 5a). Only active genes with a significant pause (Fisher’s exact test, *P*-value <0.01) were included in this analysis. We found that the PI of BEAF-associated promoters is similar but slightly lower than for M1BP-associated promoters (MWU, *P*-value = 0.0070), while the PI of GAF-associated promoters is much higher (MWU, *P*-value <2.2 × 10^−16^). Considering only promoters associated with both BEAF and M1BP, the PI was slightly higher than for all BEAF-associated genes (MWU, *P*-value = 4.3 × 10^−6^) or all M1BP-associated genes (MWU, *P*-value = 0.023). Similar consideration of promoters associated with both BEAF and GAF found that the PI was significantly higher than for all BEAF-associated genes (MWU, *P*-value <2.2 × 10^−16^) but significantly lower than for all GAF-associated genes (MWU, *P*-value = 4.7 × 10^−6^). Possible explanations for these results are that BEAF and M1BP synergize to strengthen pausing at promoters with weak pausing, while as previously proposed (Fuda *et al.* 2015; Duarte *et al.* 2016), BEAF and GAF can be antagonistic such that BEAF weakens GAF-induced pausing.

BEAF or Pbro knockdown relative to LacZ RNAi both led to a similar decrease in the PI of BEAF-associated genes (Supplementary Fig. 5; MWU, *P*-value = 2.6 × 10^−15^ and 2.9 × 10^−14^ respectively). Both also led to a decrease in the PI of genes that were not BEAF-associated, although in this case the BEAF RNAi effect was weaker and presumably indirect while Pbro RNAi had an effect similar to that at BEAF-associated genes (MWU, *P*-value = 0.0011 and 7.9 × 10^−13^ respectively). We illustrate this by comparing PI ratios, which are mostly less than 1 (Fig. 5b). For BEAF-associated genes the BEAF/LacZ RNAi PI ratio is slightly lower than the Pbro/LacZ RNAi PI ratio (MWU, *P*-value = 0.0028). For genes that are not BEAF-associated the Pbro/LacZ RNAi PI ratio is clearly lower than the BEAF/LacZ RNAi PI ratio (MWU, *P*-value <2.2 × 10^−16^). For the 2 sets of genes, the BEAF/LacZ RNAi PI ratio is clearly lower for BEAF-associated genes (MWU, *P*-value <2.2 × 10^−16^) while the Pbro/LacZ RNAi ratio is lower for genes that are not associated with BEAF (MWU, *P*-value = 1.2 × 10^−10^).

To view gene-by-gene effects on pausing we made heatmaps centered on the TSSs of the 6,069 active genes arranged in order of decreasing BEAF ChIP-seq signal (Fig. 5c). Widespread paused Pol II is apparent in the PRO-seq data for the LacZ RNAi control, while the log2 ratio plots of BEAF or Pbro to LacZ RNAi show changes after knockdown. After BEAF knockdown, there is a general decrease of promoter-proximal Pol II, even though most of this decrease is less than 1.5-fold and was not found to be significant by DESeq2. The effect is strongest for genes with the strongest BEAF signal, although the effect extends into genes with weak or no BEAF signal. A similar decrease in promoter-proximal Pol II is observed after Pbro knockdown, although the effect at BEAF-associated promoters is weaker while the effect at promoters with weak or no BEAF signal is generally stronger than after BEAF knockdown. Again, the difference is mostly less than 1.5-fold. In addition to the decrease in paused Pol II, metaplots of genes with and without a promoter-proximal BEAF peak show that gene body Pol II levels are slightly higher after BEAF or Pbro knockdown (Supplementary Fig. 5). This indicates that both treatments led to faster release from pausing rather than affecting Pol II recruitment to promoters. Like the decreases in pausing shown in Fig. 4c, these widespread decreases in pausing and PIs after BEAF or Pbro RNAi are consistent with an interaction between BEAF and Pbro slightly enhancing pausing.

### Effects of BEAF and Pbro RNAi knockdown on nucleosome positioning

**Fig. 6.**
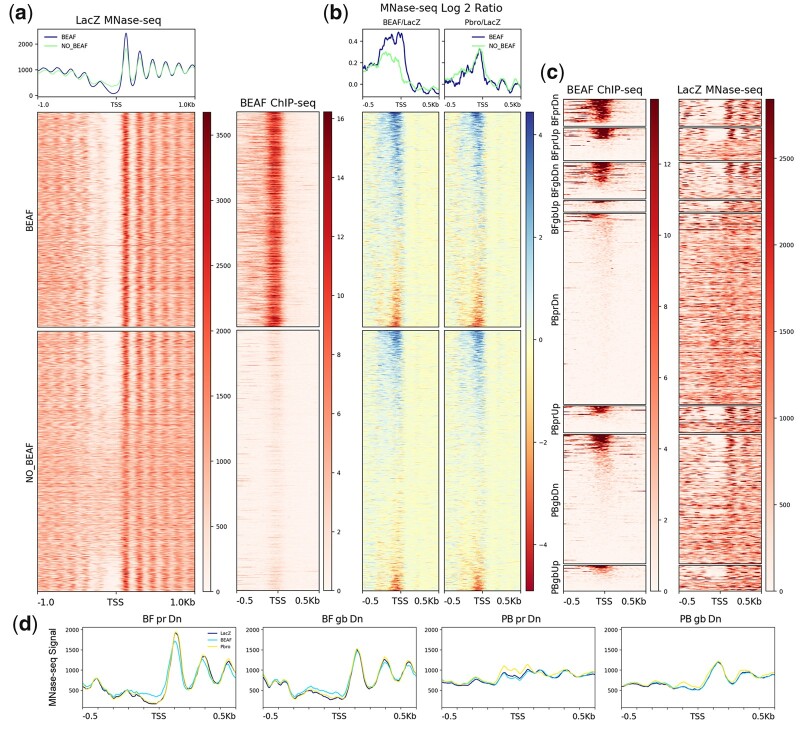
MNase-seq analysis of BEAF and Pbro RNAi knockdown effects on promoter-region nucleosomes. a) Left: Nucleosome organization after control LacZ RNAi showing 500 bp on either side of the TSS for active, paused genes with (top heatmap, *n* = 2,278) or without (bottom heatmap, *n* = 2,762) a promoter-proximal BEAF peak, sorted in order of decreasing log2(BEAF RNAi/LacZ RNAi) MNase-seq signal. Right: BEAF ChIP-seq signal arranged as in the left panel. b) Log2 heatmaps showing the promoter-region fold-change in MNase-seq signal of BEAF RNAi (left) or Pbro RNAi (right) relative to LacZ RNAi. Gene order as in (a). c) Heatmaps of differentially expressed genes arranged by promoter-region BEAF ChIP-seq signal showing the BEAF ChIP-seq signal (left) and control LacZ RNAi nucleosome organization (right). Gene numbers in each category are given in Fig. 4b. BF: BEAF RNAi; PB: Pbro RNAi; pr: promoter region; gb: gene body; Dn: downregulated; Up: upregulated. d) Promoter-region metaplots of downregulated genes for each RNAi treatment. See text for details. Labels as in (c).

Active genes have nucleosome-depleted regions (NDRs) at their TSSs. Housekeeping genes in particular have stronger NDRs and well-positioned +1 nucleosomes (Rach *et al.* 2011; Vo Ngoc *et al.* 2019). Because BEAF binds near TSSs and PBAP plays a role in remodeling chromatin during gene expression, we examined the effects of BEAF and Pbro knockdown in S2 cells using MNase-seq. Biological replicates were highly correlated (Spearman’s coefficient of 0.92 or higher for all 3 RNAi treatments in promoter and gene body regions; Supplementary Fig. 1). Active, paused genes from the LacZ RNAi control were split into genes with (BEAF) and without (no BEAF) a TSS-proximal BEAF ChIP-seq peak and sorted by decreasing log2(BEAF RNAi/LacZ RNAi) MNase-seq signal. A heatmap and associated metaplot of the control LacZ RNAi MNase-seq data show that BEAF-associated genes have stronger NDRs and stronger +1 nucleosomes than do genes lacking BEAF (Fig. 6a). This is consistent with BEAF binding near the TSS of many housekeeping genes. We also made log2 ratio MNase-seq heatmaps and metaplots of BEAF or Pbro over LacZ RNAi to highlight changes caused by these treatments (Fig. 6b). The main effect of both treatments was filling in of the NDR near a large number of TSSs, although nucleosome depletion was enhanced near a small number of TSSs (less than 10%). For BEAF RNAi, the effect was stronger for BEAF-associated genes but also occurred at genes lacking BEAF, suggesting both direct and indirect effects. For Pbro RNAi, the effect was remarkably similar to that of BEAF RNAi on a gene-by-gene basis but was weaker than for BEAF-associated genes after BEAF RNAi. In all cases the fill-in was small enough that it is hardly detected in MNase-seq metaplots (Supplementary Fig. 6). If the fill-in is due to direct effects, this is consistent with Pbro helping to recruit PBAP to many active promoters. While not conclusive, BEAF could be playing a role in this recruitment at BEAF-associated promoters.

Next, we looked at the effect of the RNAi treatments on the differentially regulated genes identified by DESeq2. Heatmaps around TSSs after control LacZ RNAi show that genes affected by BEAF RNAi, especially downregulated genes, have a strong NDR and well-positioned +1 nucleosome that correlates with the presence of BEAF (Fig. 6c). Again, this is consistent with BEAF being associated with housekeeping genes. In contrast, genes affected by Pbro RNAi generally lack promoter-proximal BEAF and have weak NDRs and poorly positioned nucleosomes, consistent with mainly affecting regulated genes. The main exception for Pbro RNAi involves the subset of genes that are BEAF-associated.

Metaplots show NDR fill-in and a shift of the +1 (and +2 and +3) nucleosome toward the TSS for genes downregulated in the promoter region after BEAF RNAi (Fig. 6d). The +1 nucleosome peak is also lower. Similar effects were reported for nonspecific lethal (NSL)-associated promoters after knockdown of NSL1 and were related to recruitment of the NURF chromatin remodeling complex, an ISWI family member (Lam *et al.* 2019). NSL1 knockdown had a stronger effect than we observed after BEAF knockdown, which is consistent with the large number of housekeeping genes showing significantly decreased expression compared to the small number of genes affected by BEAF RNAi. Genes affected by NSL1 knockdown included many associated with BEAF, M1BP, or both. The main effect on nucleosomes for genes downregulated in the gene body after BEAF RNAi or in the promoter or gene body after Pbro RNAi is slight fill-in of the NDR (Fig. 6d). In contrast, there is no clear effect on the nucleosome organization around promoters of upregulated genes after BEAF or Pbro RNAi (Supplementary Fig. 7). As with effects on transcription and pausing, effects of BEAF or Pbro RNAi on promoter-region nucleosome organization are subtle but are consistent with both proteins playing a role in gene activation.

### BEAF RNAi effects correlate better with the effects of FACT and NURF RNAi

We considered what proteins might show a better correlation with BEAF in terms of gene regulation after knockdown. Two complexes with available PRO-seq data were of particular interest: first, FACT (SSRP RNAi) (Tettey *et al.* 2019) because of its role during transcription and because both subunits were significantly enriched in our MS data (Supplementary Table 1), although we did not detect an interaction of either with BEAF by Y2H (Supplementary Fig. 8), and second, the ISWI-class chromatin remodeling complex NURF (NURF301 RNAi) (Judd *et al.* 2021). All 4 NURF subunits appear in our MS data (Supplementary Table 1), although the significance is unclear since 2 in particular were not enriched and have B&H adjusted *P*-values >0.4. NURF55, the most enriched subunit (1.4-fold, *P*-value = 0.08), is found in several protein complexes in addition to NURF. NURF can be recruited by NSL, a protein complex important for the activation of most housekeeping genes including those that are BEAF associated (Lam *et al.* 2019). We focused on promoter region effects on gene expression because BEAF binds near TSSs.

**Fig. 7.**
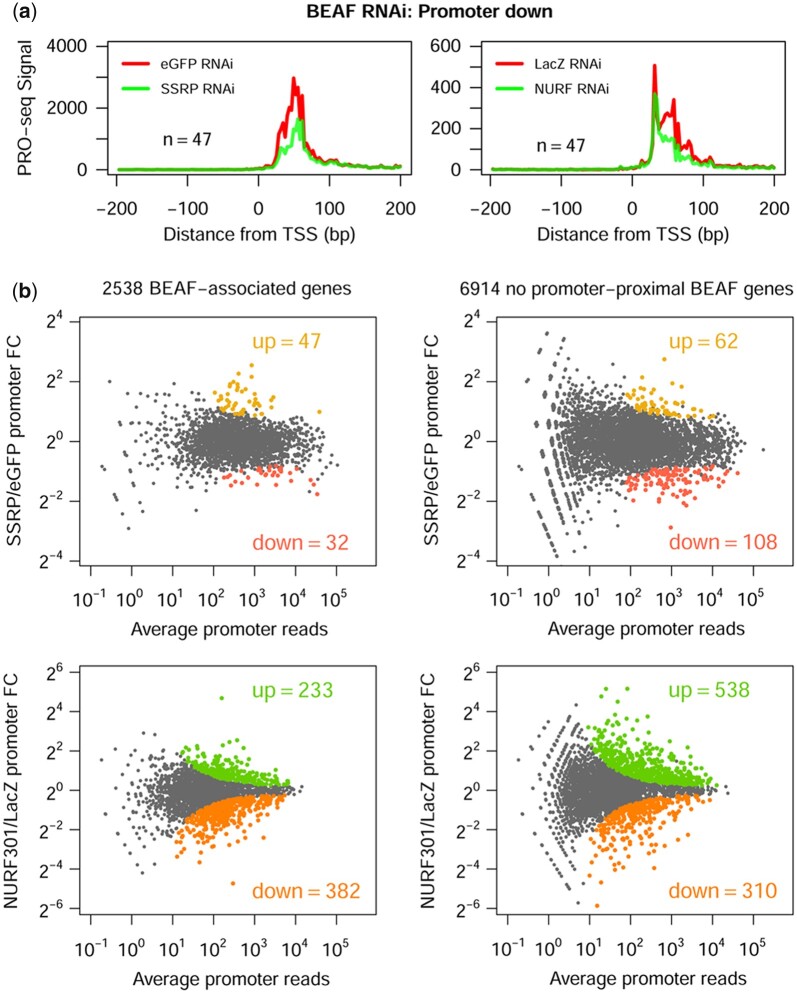
PRO-seq analysis of SSRP and NURF301 RNAi effects on transcription reveal a connection to BEAF. a) PRO-seq read density metaplots (in 3-bp bins) from −200 to +200 relative to the TSS for genes significantly downregulated in this region after BEAF knockdown, showing results of SSRP knockdown (left graph) or NURF301 knockdown (right graph). The relevant experimental and control RNAi treatments are shown in each graph for the 47 genes (*n*). The metaplots show that FACT and NURF play roles in pausing at these genes that also require BEAF for pausing. b) DESeq2 analysis of the effects of SSRP and NURF301, relative to control, RNAi on Pol II occupancy in the promoter-proximal region for BEAF-associated genes (*n* = 2,538) and genes lacking a BEAF ChIP-seq peak within 500 bp of the TSS (*n* = 6,914). MA plots are shown with significantly upregulated and downregulated genes, defined as having an FDR of 0.05 or less, indicated by colored dots and numbers. FC: fold change. Compared to all genes, SSRP upregulated genes and NURF301 downregulated genes are enriched for BEAF-associated genes (Fisher’s exact test, *P*-value = 0.0101 and <0.0001, respectively).

Genes with significantly decreased promoter-proximal Pol II pausing after BEAF RNAi also show decreased pausing after SSRP or NURF301 RNAi (Fig. 7a). NURF RNAi also caused the pause to shift toward the TSS, as previously reported (Lam *et al.* 2019). As for Pbro, this set of genes showed the best correlation between BEAF knockdown and SSRP or NURF301 knockdown (Supplementary Fig. 9). This suggests that genes that are most dependent on BEAF for Pol II pausing also depend on recruitment of PBAP, NURF, and FACT. In addition, unlike Pbro RNAi, genes whose promoter-proximal Pol II pausing is affected by NURF301 or SSRP knockdown show a correlation with BEAF-associated genes (Fig. 7b). For NURF301 RNAi the correlation is with genes with decreased pausing (381 BEAF-associated genes of 690 total genes called as differentially regulated by DESeq2 with a *P*adj of <0.05), suggesting BEAF could work with NURF to stabilize pausing at these genes or enhance pause release in the absence of NURF. For SSRP RNAi the correlation is with genes with increased pausing (47 BEAF-associated genes of 109 total genes called as differentially regulated by DESeq2 with a *P*adj of <0.05), suggesting BEAF could work with FACT to promote pause release at these genes or stabilize pausing in the absence of FACT. Full DESeq2 results are found in Supplementary Table 6. The effect of potential interactions between BEAF and FACT may be context dependent since at genes most dependent on BEAF for pausing, BEAF and FACT both promote pausing (Fig. 7a). This analysis supports the idea that redundant factors are at play at BEAF-associated genes, with FACT and especially NURF playing larger roles than PBAP.

## Discussion

### A closer look at the interaction between BEAF and Pbro

The roles of BEAF in promoter and insulator function can be separated (Maharjan *et al.* 2020). To gain insight into molecular mechanisms of BEAF function, we are identifying proteins that physically interact with BEAF. Here, we provide evidence that BEAF physically interacts with the Pbro subunit, and genetically interacts with several subunits, of the PBAP chromatin remodeling complex. An interaction between BEAF and PBAP is also supported by a high-throughput screen that transfected epitope-tagged DNA-binding proteins into S2 cells followed by identification of copurifying proteins via mass spectrometry (Rhee *et al.* 2014). In that study, BEAF pulled down the PBAP-specific subunit Bap170, the BAP-specific subunit Osa, and all proteins in common between PBAP and BAP except Brm. Although Pbro was not detected in these experiments, overall the data provide supporting evidence that PBAP and possibly BAP interact with BEAF either directly or indirectly.

Pbro has 6 BDs, and the original Y2H interaction we detected was with the region from BD2 to BD5 or BD6. Y2H indicated that single BDs do not interact with BEAF, and the presence of BD2 might be inhibitory. The best interactions were obtained with BD3–4 and BD4–5 pairs, a result mirrored in the pulldown experiments. It was shown for the human Pbro homolog PBRM1 that BD2 and BD4 are critical for binding to nucleosomes, favoring binding to acetylated histone H3 lysine 14 or trimethylated H3 lysine 4 (Slaughter *et al.* 2018). While there could be functional differences between PBRM1 and Pbro, there is high sequence homology between equivalent human and *Drosophila* BDs, with 69% similarity in BD4. It is possible that BEAF influences the interaction of PBAP with nucleosomes by interacting with the BD4 region of Pbro.

Y2H indicated the putative LZ plus the middle region of BEAF is important for the Pbro interaction. The 40 amino acids immediately upstream of the LZ are not highly conserved among *Drosophila* species. We previously found that a *BEAF* transgene lacking this nonconserved region can rescue a null *BEAF* mutation, and a transgene lacking the LZ can also rescue but is hypomorphic (Avva and Hart 2016). This suggests the interaction between BEAF and PBAP might improve health but is not essential, a point supported by our PRO-seq results that found broad weak effects but minimal overlap in genes significantly affected by BEAF or Pbro knockdown. This is consistent with our Y2H results that we interpret as indicating the interaction is weak or requires a specific condition such as an unusual conformation or posttranslational modification of 1 or both interacting surfaces. BEAF is known to be phosphorylated and glycosylated (Hart *et al.* 1997; De *et al.* 2018), although whether this happens in yeast is unknown. In addition to gene regulation, PBAP has been implicated in insulator activity (Nakayama *et al.* 2012). Although other explanations for healthier flies when BEAF has the LZ cannot be excluded, maybe the BEAF–Pbro interaction fine-tunes gene expression or insulator activity to contribute to fly homeostasis.

### Perspective on effects of BEAF knockdown on transcription and chromatin

Knockdown of BEAF had a significant effect on a small number of genes relative to the number of genes with a promoter-proximal BEAF ChIP-seq peak. Of these, most that are BEAF-associated (likely to be a direct effect) are downregulated. This is consistent with reports that BEAF is mainly associated with gene activation (Emberly *et al.* 2008; Jiang *et al.* 2009; Dong *et al.* 2020; Maharjan *et al.* 2020). It is also consistent with the limited effects of a null mutation. Maternal BEAF is sufficient for development of *BEAF* null flies whose most obvious defect is near infertility of females, indicating BEAF is only essential during oogenesis and/or early embryogenesis (Roy, Gilbert, *et al.* 2007). In fact, we have uncovered an unidentified chromosome 2 dominant mutation we call *Tofu* that restores fertility to females, allowing us to maintain stocks lacking BEAF (Hart 2014). This implies there are redundant factors that can work without BEAF to maintain promoter and insulator function. One candidate is M1BP (Li and Gilmour 2013), which colocalizes with BEAF at several hundred promoter regions. Another candidate is the NSL complex, which localizes to the promoter region of a large number of housekeeping genes and plays an important role in their regulation (Lam *et al.* 2012); like BEAF (Emberly *et al.* 2008; Maharjan *et al.* 2020), it binds to AT-rich promoter regions (Lam *et al.* 2019). NSL colocalizes to over 90% of BEAF-associated promoter regions (and over 90% of M1BP-associated promoters). NSL has been shown to recruit the NURF chromatin remodeling complex (Lam *et al.* 2019).

BEAF-associated promoters are nucleosome-depleted upstream of TSSs and have a well-positioned +1 nucleosome, characteristics of housekeeping genes (Rach *et al.* 2011; Lam *et al.* 2012). After BEAF knockdown, the 47 genes showing a significant decrease in Pol II promoter-proximal pausing had an increase in nucleosome occupancy of the promoter NDR together with a shift of the +1 nucleosome toward the TSS. Most of these genes were BEAF associated (87%). Consistent with the possibility that BEAF and NSL play redundant roles with NSL being more important, this is similar to what was observed at most NSL-associated promoters after NSL1 knockdown (Lam *et al.* 2019). M1BP is also found near the TSS of many housekeeping genes and, like M1BP-associated genes (Li and Gilmour 2013), we found that BEAF-associated genes have relatively weak promoter-proximal Pol II pausing. This is consistent with pausing of BEAF-associated genes being due to the energetic barrier of Pol II penetrating into the +1 nucleosome (Bintu *et al.* 2012; Gaykalova *et al.* 2015), as previously proposed for M1BP-associated genes (Li and Gilmour 2013). Combined with the additive effect on Pol II pausing at promoters associated with both BEAF and M1BP, it is likely that both proteins contribute to pausing in similar ways. For instance, both could help recruit NSL or help position and stabilize the +1 nucleosome to slightly impair the ability of Pol II to overcome this barrier. In contrast, pausing is reduced at promoters associated with GAF if BEAF is also present. GAF recruits NELF to enhance pausing by Pol II before the +1 nucleosome is reached (Li *et al.* 2013). BEAF might antagonize GAF-mediated pausing by interfering with this recruitment.

### Perspective on effects of Pbro knockdown on transcription and chromatin

PBAP is recruited to chromatin by other proteins rather than binding a specific DNA sequence. The stable association of Pbro and Bap170 in the PBAP complex requires the SAYP subunit (Chalkley *et al.* 2008), suggesting that a partial PBAP complex can form without Pbro. Work with vertebrates found that the homologous PBAF complex is necessary for ligand-induced transcription activation by nuclear receptors (Lemon *et al.* 2001), and the histone acetyltransferases CBP/p300 can recruit PBAF/BAF (Huang *et al.* 2003). CBP/p300 are recruited to active promoters and enhancers in flies and vertebrates (Wang *et al.* 2009; Philip *et al.* 2015) by a large number of proteins (Goodman and Smolik 2000; Bedford *et al.* 2010). Targeting the PBAP subunit Bap170 to promoter regions can facilitate gene activation by distant enhancers (Shidlovskii *et al.* 2021). Combined with evidence that PBAF and CBP/p300 can help Pol II overcome the +1 nucleosome barrier (Nock *et al.* 2012; Boija *et al.* 2017), this suggests that promoter-proximal PBAP should play a role in gene activation. In agreement with this, we found that Pbro knockdown mainly downregulates gene expression. These genes were not enriched for BEAF-associated genes, indicating that the interaction between BEAF and Pbro does not have a large effect on gene regulation in S2 cells. Instead, we found that these genes were enriched for GAF-associated promoters, in line with other reports (Nakayama *et al.* 2012; Judd *et al.* 2021).

Pbro knockdown did not have much effect on promoter-region chromatin of genes whose expression was significantly altered by the knockdown. There was some fill-in of the promoter NDR. However, MNase-seq did find that most genes whose expression was altered by Pbro knockdown lack a well-defined promoter NDR and do not have a well-positioned +1 nucleosome. This is consistent with Pbro playing a larger role in the expression of regulated genes, including those where GAF plays a role.

### Further exploration of the interaction between BEAF and Pbro

Our results indicate that BEAF and Pbro physically and genetically interact, but this interaction only plays a subtle role in gene regulation in S2 cells. The PRO-seq and MNase-seq experiments most clearly provide insight into the separate roles of BEAF and Pbro in gene expression, pausing, and promoter-region nucleosome organization. They confirm and extend previous studies showing BEAF binds near housekeeping gene TSSs (Bushey *et al.* 2009; Jiang *et al.* 2009; Nègre *et al.* 2010), while PBAP plays a larger role in the expression of regulated genes including GAF-associated genes (Nakayama *et al.* 2012; Judd *et al.* 2021). But they are also consistent with the interaction between BEAF and Pbro having a minor influence on Pol II pausing and promoter NDR nucleosome occupancy. Combined with previous results showing that BEAF can activate certain promoters (Dong *et al.* 2020; Maharjan *et al.* 2020), they also suggest that functional redundancy could mask the role of BEAF at housekeeping promoters. We identified FACT and NURF as potential redundant factors that might also directly or indirectly interact with BEAF to mask the effect of an interaction with PBAP. PRO-seq data obtained after SSRP or NURF301 RNAi (Tettey *et al.* 2019; Judd *et al.* 2021) showed a better correlation with our BEAF RNAi PRO-seq data and with BEAF-associated genes than did Pbro RNAi. PBAP, FACT, and NURF can be recruited by multiple proteins, supporting the idea that BEAF could be part of a network of interactions with built in redundancy to assure essential genes remain active. Two approaches might help demonstrate the importance of the interaction between BEAF and Pbro as well as other factors. One approach would use combinatorial knockdowns to determine the importance of potential regulatory partners in the regulation of BEAF-associated genes. The other would be to use conditions where the role of BEAF is most important, such as during oogenesis or early embryogenesis (Roy, Gilbert, *et al.* 2007). Perhaps after the maternal-to-zygotic switch in gene expression, redundant mechanisms keep BEAF-associated genes active and independently recruit PBAP or other chromatin remodelers. Future experiments are needed to determine the significance of the interaction between BEAF and the Pbro subunit of PBAP, as this will help understand molecular mechanisms by which BEAF affects gene expression.

## Supplementary Material

jkac223_FiguresS1-S9-TableS2

jkac223_TableS1

jkac223_TableS3

jkac223_TableS4

jkac223_TableS5

jkac223_TableS6

## Data Availability

Strains and plasmids are available upon request. Supplementary Table 1: coimmunoprecipitation tandem mass spectrometry results. Supplementary Table 2: sequencing and alignment parameters. Supplementary Fig. 1: biological replicate correlation plots. Supplementary Fig. 2: SEM images of rough eye genetic assay negative results. Supplementary Fig. 3: full Western blots used to make Fig. 4a. Supplementary Fig. 4: differentially expressed gene PRO-seq metaplots, promoter, and gene body regions. Supplementary Fig. 5: BEAF-associated and no BEAF active, paused gene PRO-seq metaplots, promoter, and gene body regions. Supplementary Fig. 6: BEAF-associated and no BEAF active, paused gene MNase-seq metaplots and promoter regions. Supplementary Fig. 7: differentially expressed gene MNase-seq metaplots, promoter regions. Supplementary Fig. 8: Y2H results of testing for interactions between BEAF-32B and FACT subunits. Supplementary Fig. 9: differentially expressed gene PRO-seq metaplots, promoter and gene body regions after BEAF, SSRP, or NURF301 RNAi. Supplementary Table 3: DESeq2 results for BEAF and Pbro RNAi. Supplementary Table 4: overlaps between different categories of differentially expressed genes after BEAF and Pbro RNAi. Supplementary Table 5: pausing index analysis after LacZ, BEAF, and Pbro RNAi. Supplementary Table 6: DESeq2 results for SSRP and NURF301 RNAi. PRO-seq and MNase-seq data are available at GEO with the accession number: GSE197584. Custom code used to process data can be found at https://github.com/McKowen-JK/BEAF_Pbro. Supplemental material is available at G3 online.
