# Supplementary material for: The Drosophila BEAF insulator protein interacts with the polybromo subunit of the PBAP chromatin remodeling complex
Source: G3 (Bethesda). 2022 Aug 27;12(11):jkac223. doi: 10.1093/g3journal/jkac223 (PMC9635645; doi:10.1093/g3journal/jkac223)
Supplement: jkac223_FiguresS1-S9-TableS2 [file jkac223_figuress1-s9-tables2.pdf]

A

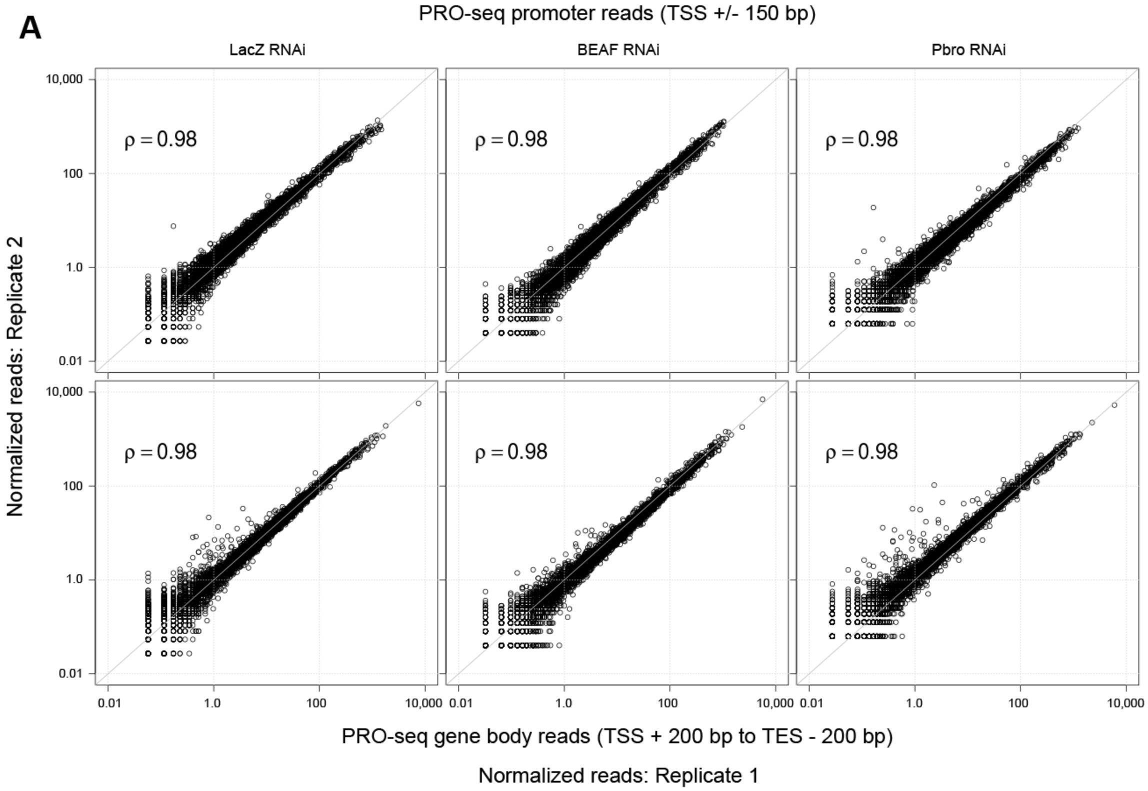
PRO-seq promoter reads (TSS +/- 150 bp)
LacZ RNAi
BEAF RNAi
Pbro RNAi
ρ = 0.98
ρ = 0.98
ρ = 0.98
ρ = 0.98
ρ = 0.98
ρ = 0.98
Normalized reads: Replicate 2
10,000
100
1.0
0.01
0.01
1.0
100
10,000
PRO-seq gene body reads (TSS + 200 bp to TES - 200 bp)
Normalized reads: Replicate 1

B

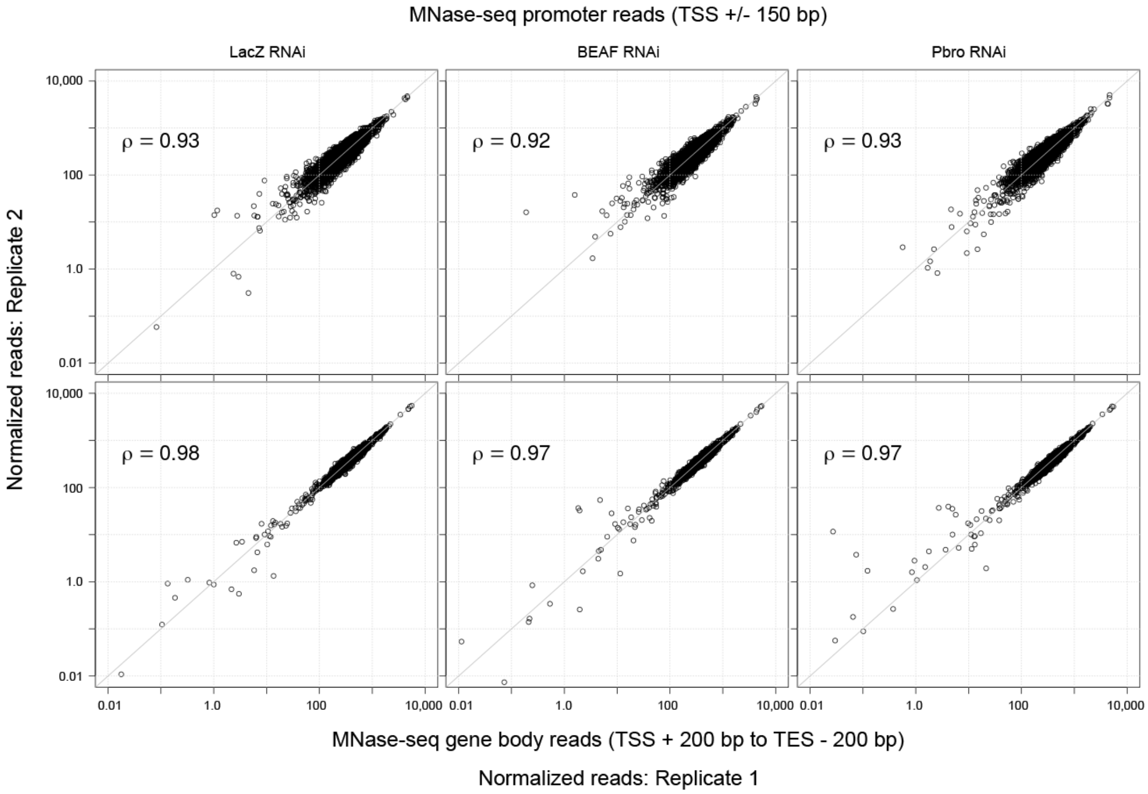
MNase-seq promoter reads (TSS +/- 150 bp)
LacZ RNAi
BEAF RNAi
Pbro RNAi
ρ = 0.93
ρ = 0.92
ρ = 0.93
ρ = 0.98
ρ = 0.97
ρ = 0.97
Normalized reads: Replicate 2
10,000
100
1.0
0.01
0.01
1.0
100
10,000
MNase-seq gene body reads (TSS + 200 bp to TES - 200 bp)
Normalized reads: Replicate 1

Figure S1. Biological replicates of PRO-seq and MNase-seq libraries are highly correlated. (A) Correlation plots of PRO-seq reads of 9452 genes in the promoter regions (upper plots; 150 bp on either side of the annotated TSS) and gene body regions (lower plots; from 200 bp downstream of the TSS to 200 bp upstream of the annotated gene end, or transcription end site (TES)) of biological replicates for each RNAi treatment (LacZ, BEAF and Pbro). The gray diagonal lines represent a 1:1 correspondence. Spearman's correlation coefficients are shown in the plots. (B) Correlation plots of MNase-seq reads of 9452 genes in the promoter regions (upper plots) and gene body regions (lower plots) of biological replicates for each RNAi treatment (LacZ, BEAF and Pbro). The gray diagonal lines represent a 1:1 correspondence. Spearman's correlation coefficients are shown in the plots.

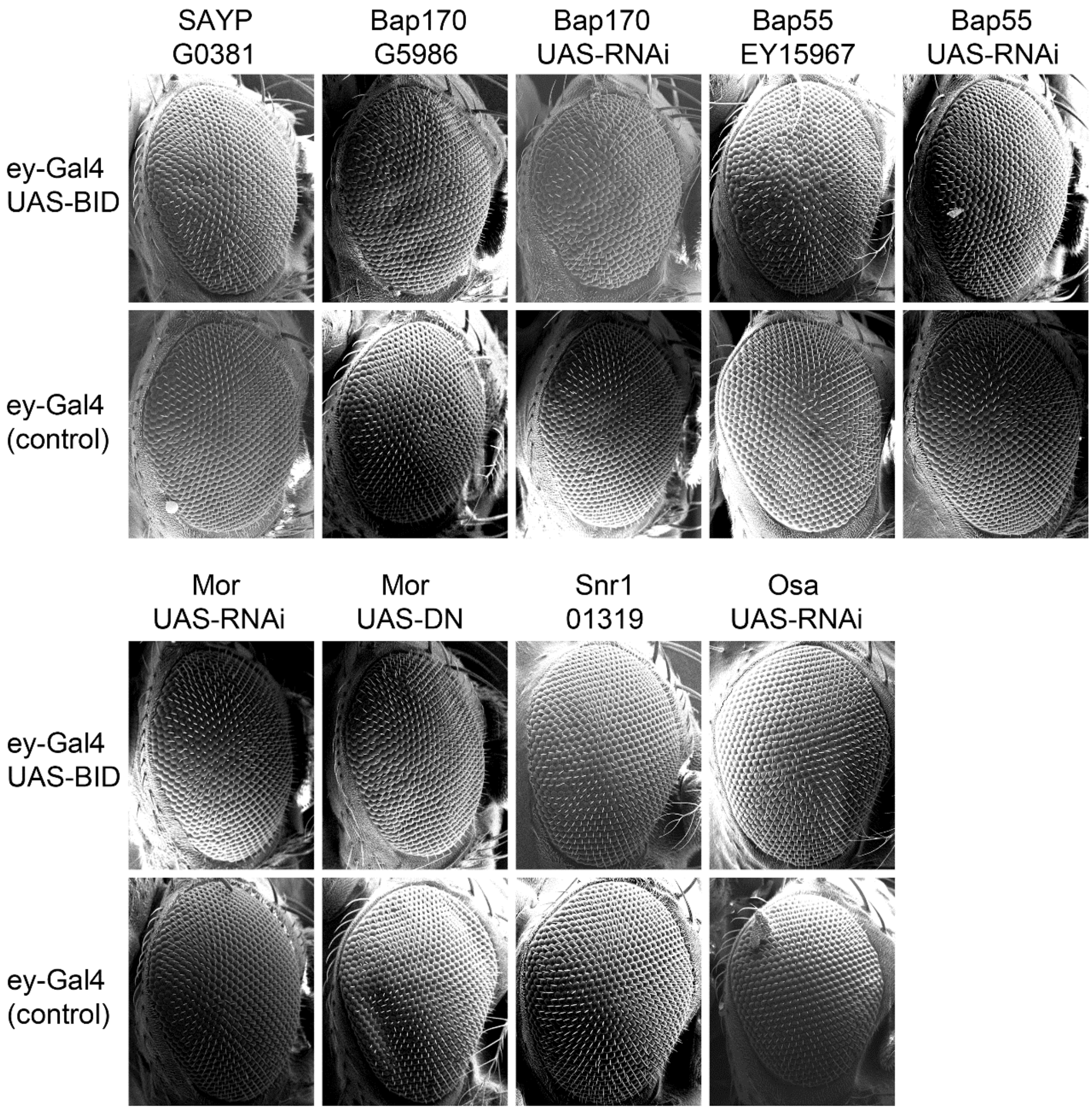

Figure S2. PBAP lines that did not show a genetic interaction with BEAF. A heterozygous *UAS-BID* BEAF dominant negative transgene causes a mild rough eye phenotype when driven by a heterozygous *ey-Gal4* transgene (not shown). SEM images of eyes also heterozygous for *UAS-RNAi*, *UAS-DN* (dominant negative), or a mutation affecting the indicated genes do not show an enhanced rough eye phenotype (*ey-Gal4*, *UAS-BID* panels). They might show suppression, but this was not scored as the starting rough eye phenotype is mild. Control panels lack *UAS-BID*.

Image Display Parameters

Channel	Color	Minimum	Maximum	K
700	Red	9.20E1	4.98E3	0
800	Green	1.80E1	4.98E3	0

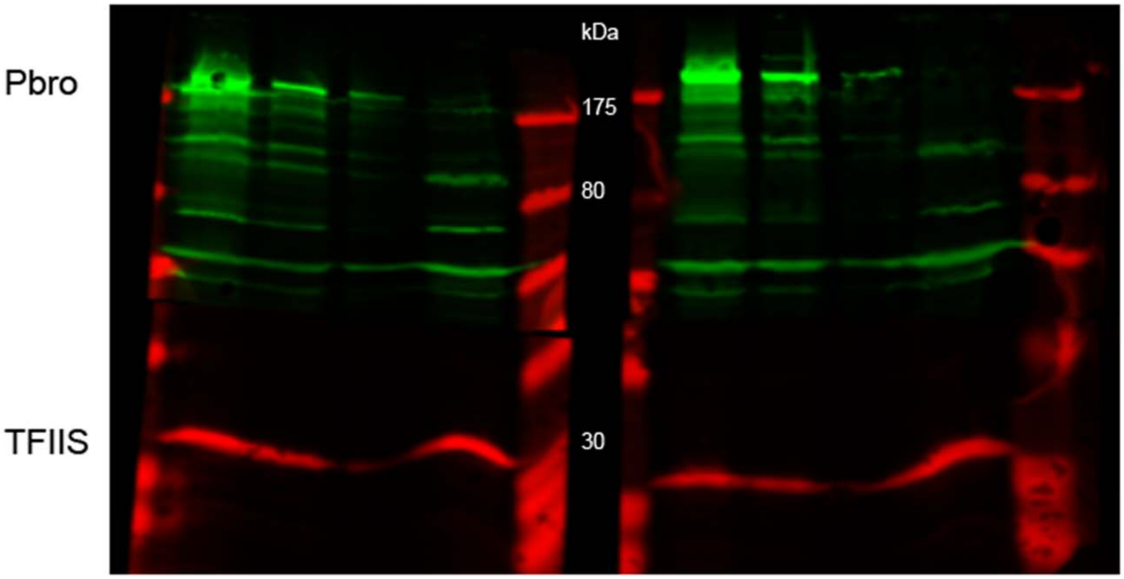

Image Display Parameters

Channel	Color	Minimum	Maximum	K
700	Red	5.96E-8	8.58E3	0
800	Green	6.00E0	5.92E3	0

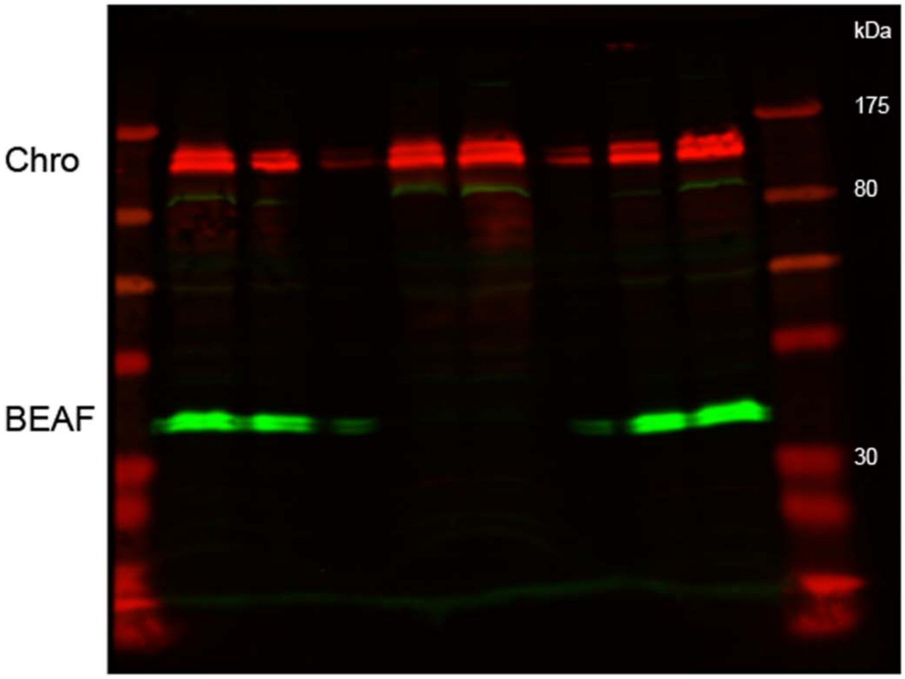

Figure S3. Western blots used to make Figure 4A. See Materials and Methods for a description.

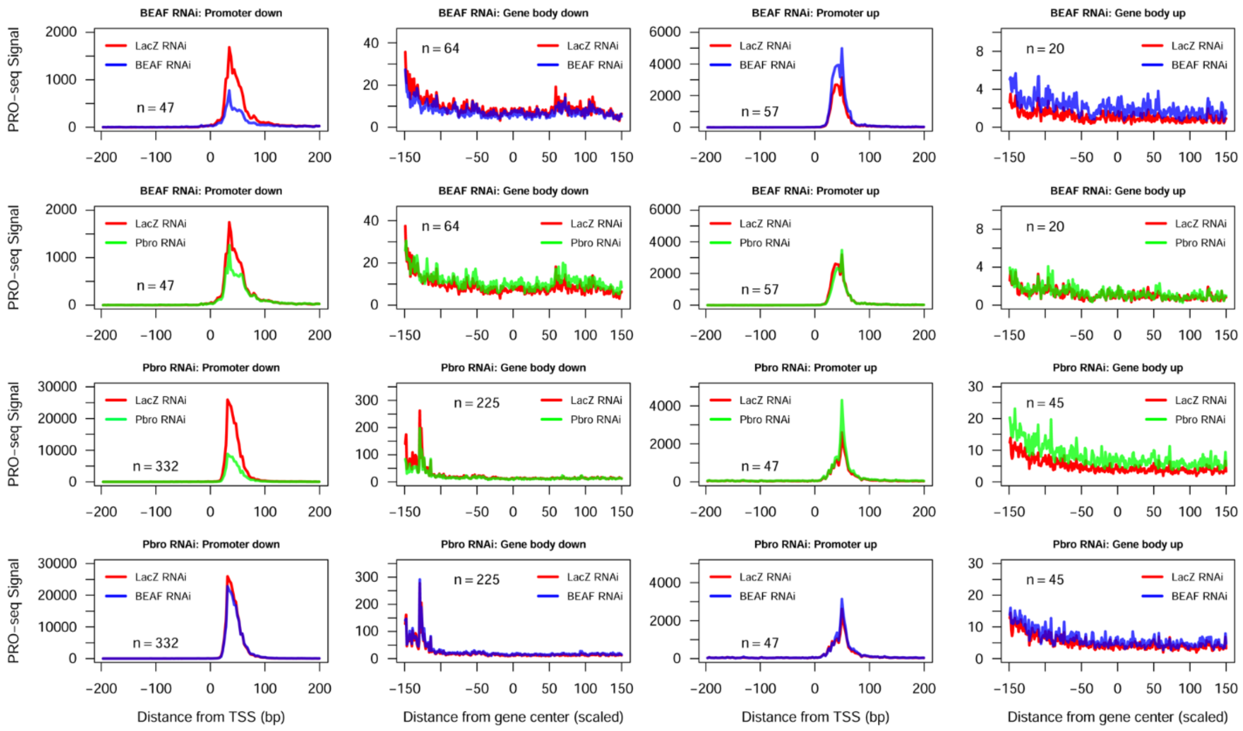

Figure S4. PRO-seq read density metaplots of differentially expressed genes. DESeq2 was used with an FDR of 0.05 to identify genes significantly differentially regulated in the promoter region (50 bp window with the most reads from -50 to 150 relative to the annotated TSS) or gene body (from 200 bp downstream of the TSS to 200 bp upstream of the annotated gene end) in the experimental condition relative to control LacZ RNAi. The experimental condition and DESeq2 result (promoter or gene body, up or down-regulated relative to LacZ RNAi) is given above each metaplot, with the number of genes affected (n) and the displayed RNAi treatments indicated in the plot. Graphs for the promoter regions (200 bp on either side of the TSS) and gene bodies (scaled to the same length) are shown for genes differentially regulated in either the promoter region or gene body, as indicated. The strongest effect is seen in the promoter region for genes down-regulated in the promoter region. The strongest correlation between BEAF and Pbro RNAi is for genes downregulated in the promoter region after BEAF RNAi.

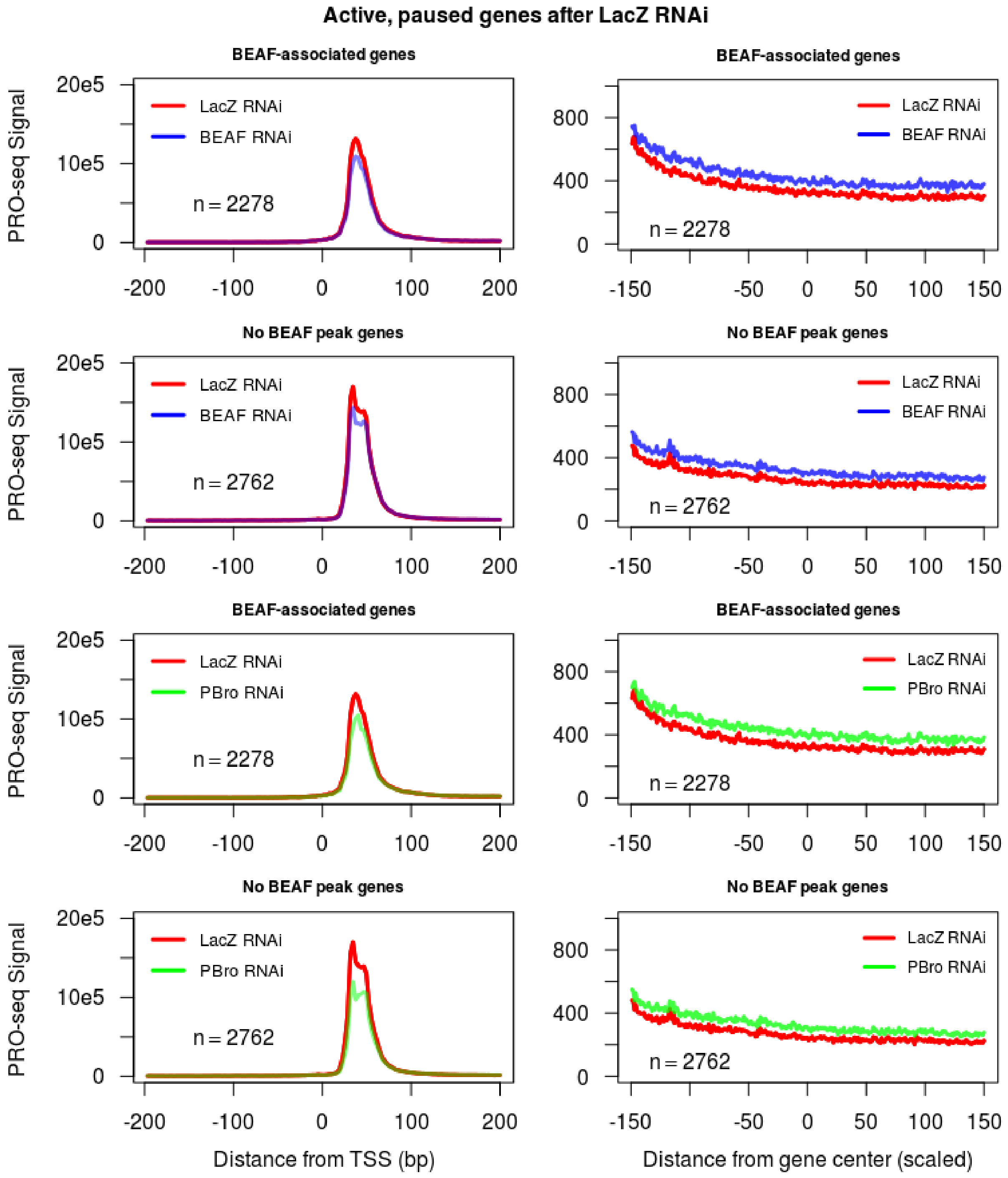
Active, paused genes after LacZ RNAi
BEAF-associated genes
No BEAF peak genes
PRO-seq Signal
20e5
10e5
0
800
400
LacZ RNAi
BEAF RNAi
PBro RNAi
n = 2278
n = 2762
-200
-100
100
200
-150
-50
50
150
Distance from TSS (bp)
Distance from gene center (scaled)

Figure S5. PRO-seq read density metaplots of active, paused genes with or without a promoter-proximal BEAF ChIP-seq peak. Active (over 50 reads when combining promoter-proximal and gene body reads for both replicates), paused (promoter-proximal Pol II density divided by the gene body Pol II density; Fisher's exact test, P-value < 0.01) genes after control LacZ RNAi treatment were extracted from 9452 genes and split into genes with a BEAF ChIP-seq peak apex within 500 bp of the annotated TSS and those lacking a BEAF peak, as indicated above the metaplots. Metaplots of the promoter regions (200 bp on either side of the TSS) and gene bodies (from 200 bp downstream of the TSS to 200 bp upstream of the annotated gene end, scaled to the same length) were made for both gene lists comparing the indicated experimental condition (BEAF or Pbro RNAi) to the control LacZ RNAi. The number of genes is indicated in the plots (n). On average, there was a slight decrease in promoter proximal Pol II and a slight increase in gene body Pol II for both sets of genes after either experimental treatment. This indicates the changes are due to reduced promoter-proximal Pol II pausing rather than Pol II recruitment.

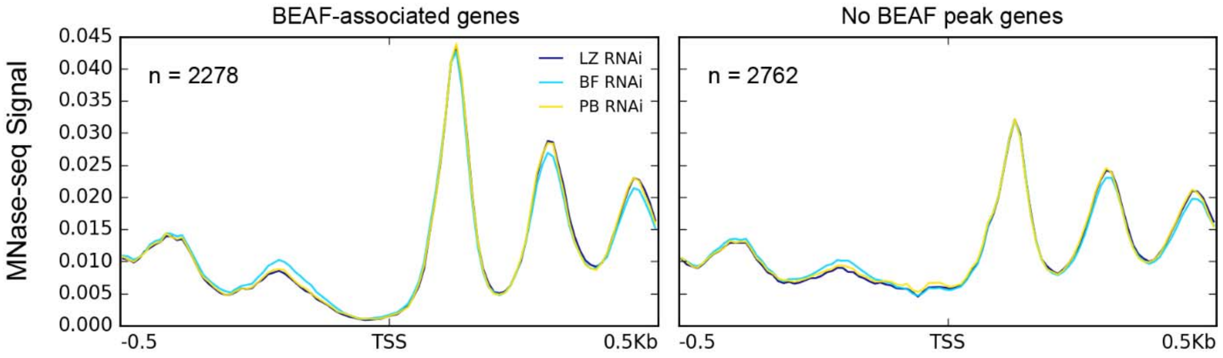

Figure S6. MNase-seq nucleosome center metaplots of active, paused genes with or without a promoter-proximal BEAF ChIP-seq peak. Active (over 50 PRO-seq reads when combining promoter-proximal and gene body reads for both replicates), paused (PRO-seq promoter-proximal Pol II density divided by the gene body Pol II density; Fisher's exact test, P-value < 0.01) genes after control LacZ RNAi treatment were extracted from 9452 genes and split into genes with a BEAF ChIP-seq peak apex within 500 bp of the annotated TSS and those lacking a BEAF peak, as indicated above the metaplots. Metaplots (500 bp on either side of the TSS) of nucleosome centers were made for both gene lists comparing the experimental conditions (BEAF and Pbro RNAi) to the control LacZ RNAi. The number of genes is indicated in the plots (n). This bulk view of promoter regions for these two categories of genes revealed only slight differences in nucleosome occupancy and no difference in nucleosome positioning, even for BEAF-associated genes after BEAF knockdown. However, it is clear that BEAF-associated genes have a stronger NDR and higher +1 nucleosome occupancy.

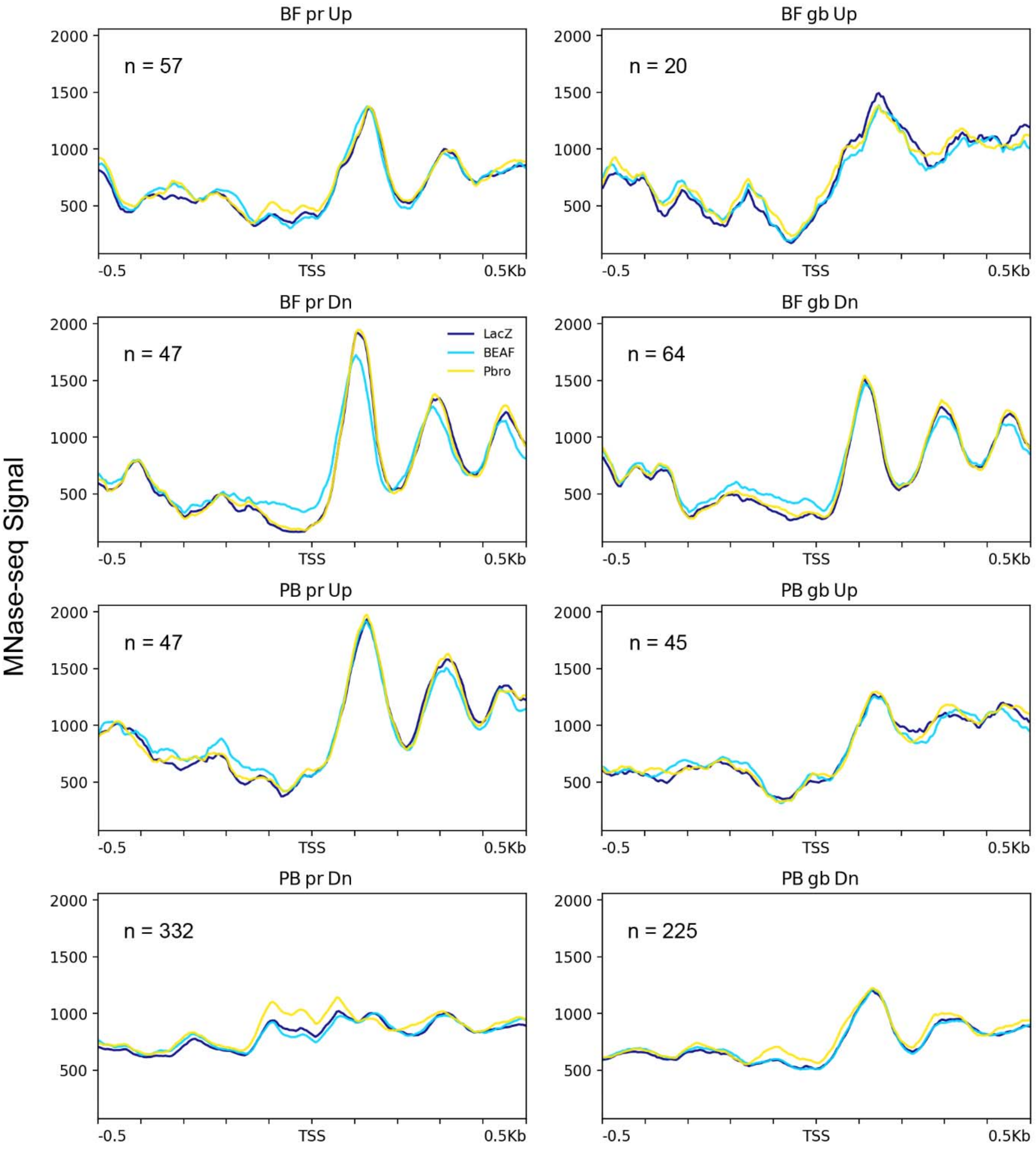

Figure S7. Metaplots of MNase-seq nucleosome centers of differentially expressed genes. DESeq2 was used with PRO-seq data with an FDR of 0.05 to identify genes significantly differentially regulated in the promoter region (50 bp window with the most reads from -50 to 150 relative to the annotated TSS) or gene body (from 200 bp downstream of the TSS to 200 bp

upstream of the annotated gene end) in the experimental condition relative to control LacZ RNAi. The DESeq2 result relative to LacZ RNAi is given above each metaplot (BF pr Dn: promoter-proximal Pol II down after BEAF RNAi; BF pr Up: promoter-proximal Pol II up after BEAF RNAi; BF gb Dn: gene body Pol II down after BEAF RNAi; BF gb Up: gene body Pol II up after BEAF RNAi; PB pr Dn: promoter-proxima Pol II down after Pbro RNAi; PB pr Up: promoter-proximal Pol II up after Pbro RNAi; PB gb Dn: gene body Pol II down after Pbro RNAi; PB gb Up: gene body Pol II up after Pbro RNAi), with the number of genes affected indicated in the plot (n). Metaplots show nucleosome centers (500 bp on either side of the TSS) of the experimental (BEAF and Pbro RNAi) and control LacZ RNAi conditions for genes differentially regulated in either the promoter region or gene body, as indicated. The clearest effects are seen for genes down-regulated in the promoter region. BEAF knockdown results in increased nucleosome occupancy in the NDR and slight decrease in nucleosome occupancy and shift towards the TSS of the +1, +2 and +3 nucleosomes for genes downregulated in this region after BEAF knockdown. Pbro knockdown results in increased nucleosome occupancy in the NDR and immediately downstream of the TSS for genes downregulated in this region after Pbro knockdown, although the NDR is weaker and downstream nucleosomes are not well positioned at these genes compared to genes downregulated in this region by BEAF RNAi.

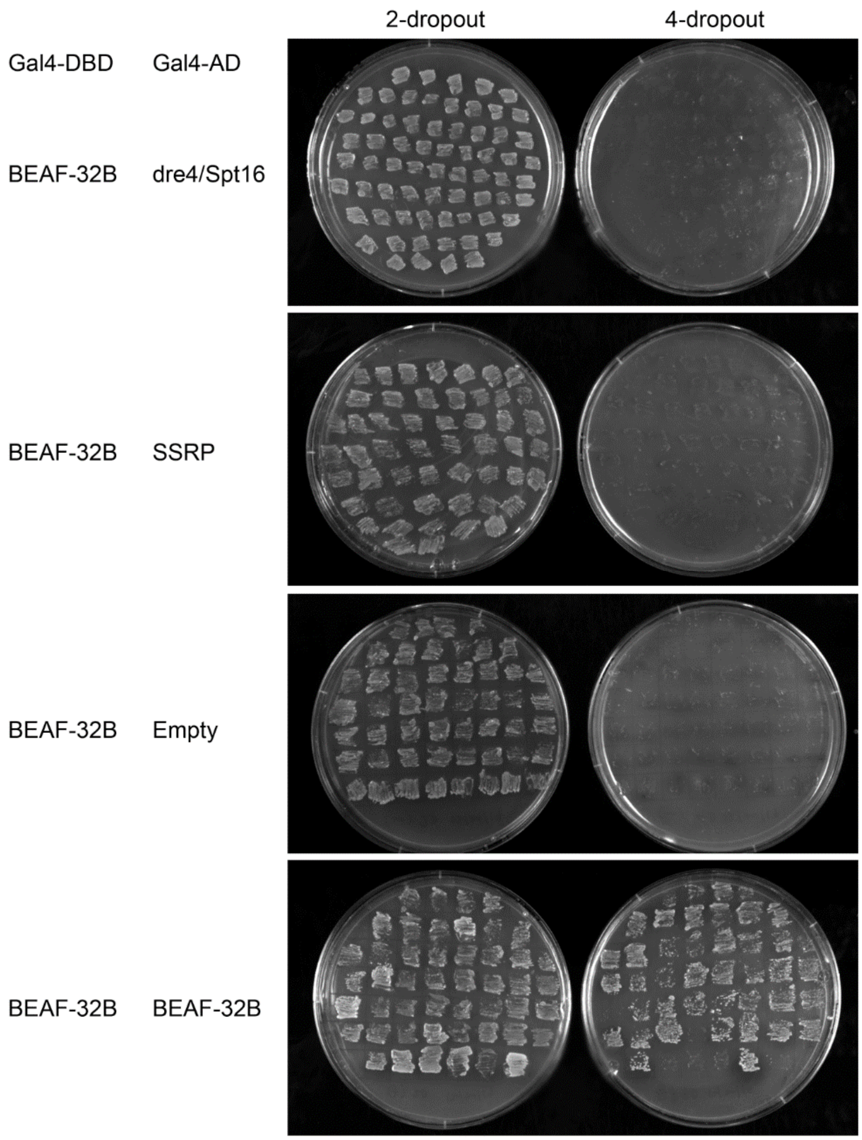

Figure S8. Y2H results do not show an interaction between FACT subunits and BEAF. Approximately 50 colonies are shown for each combination. Yeast grew on 2-dropout plates showing the presence of the Gal4-DBD-BEAF-32B and indicated Gal4-AD-cDNA plasmids. Yeast did not grow on 4-dropout plates for either FACT subunit (dre4: DGRC clone AT29108; SSRP: DGRC clone FI07619) or the empty vector control, indicating no interaction with BEAF-32B. Yeast grew on 4-dropout plates for the positive control, BEAF-32B interacting with itself.

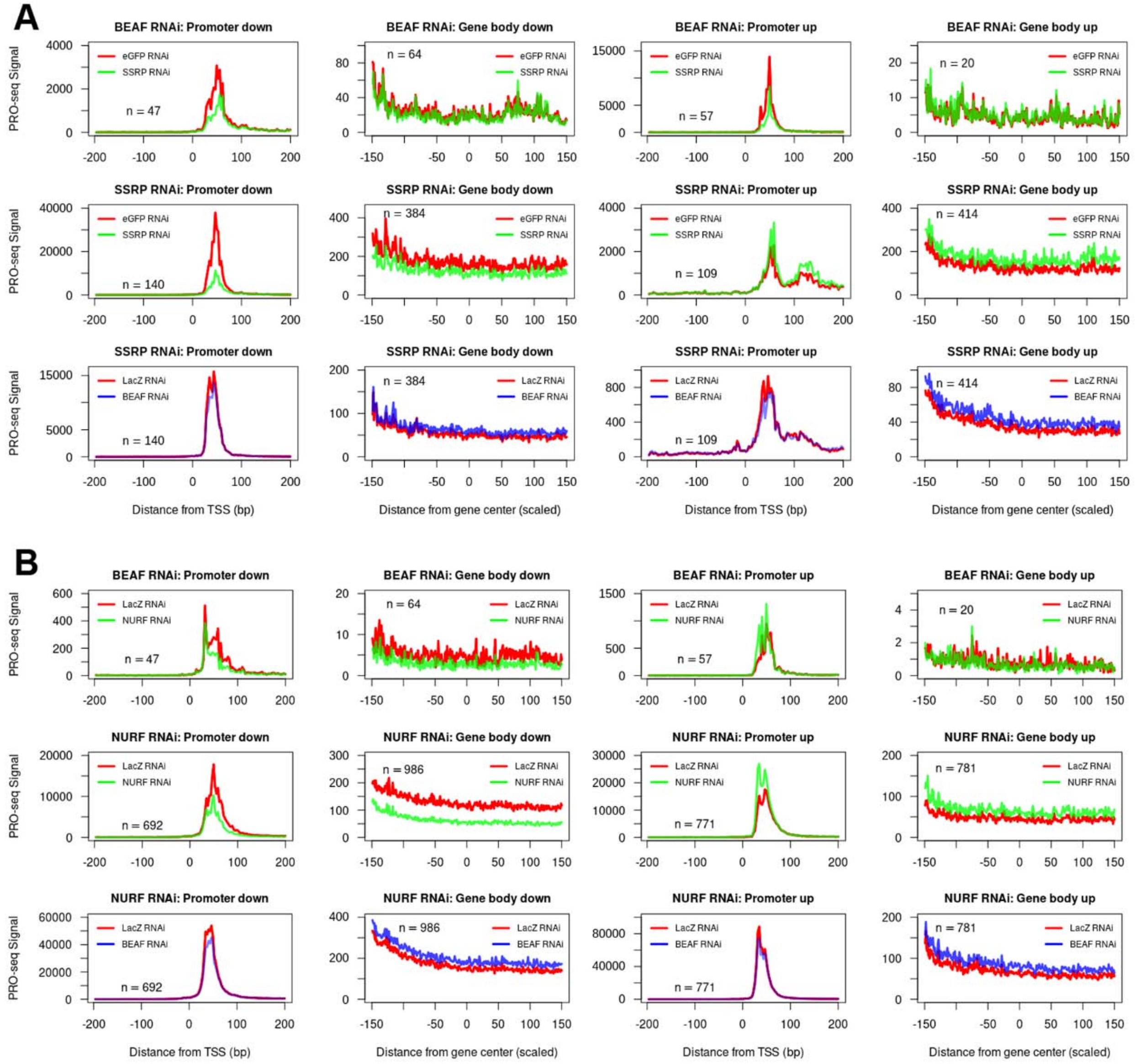

Figure S9. PRO-seq read density metaplots of differentially expressed genes comparing BEAF, FACT (SSRP) and NURF (NURF301) RNAi treatments. DESeq2 was used with an FDR of 0.05 to identify genes significantly differentially regulated in the promoter region (50 bp window with the most reads from -50 to 150 relative to the annotated TSS) or gene body (from 200 bp downstream of the TSS to 200 bp upstream of the annotated gene end) in the experimental condition relative to control LacZ or eGFP RNAi. The experimental condition and DESeq2 result (promoter or gene body, up or down-regulated relative to control RNAi) is given above each

metaplot, with the number of genes affected (n) and the displayed RNAi treatments indicated in the plot. Graphs for the promoter regions (200 bp on either side of the TSS) and gene bodies (scaled to the same length) are shown for genes differentially regulated in either the promoter region or gene body, as indicated. The strongest effects are seen for genes down-regulated in the promoter region for all experimental RNAi treatments, and down-regulated in the gene body after NURF301 knockdown. The strongest correlation between BEAF and SSRP or NURF301 RNAi is for genes downregulated in the promoter region after BEAF RNAi (see Figure S2 for the effects of BEAF RNAi on genes differentially expressed after BEAF RNAi).

Table S2: Sequencing and alignment of PRO-seq and MNase-seq libraries

Library	Total reads	Filtered, clipped	Non-ribosomal	Mapped reads	
LZ_RNAi_1	40,280,738	32,962,838	28,561,180	18,320,010	
LZ_RNAi_2	104,519,561	84,914,751	70,027,166	39,191,309	
BF_RNAi_1	78,791,075	64,940,746	53,484,121	33,124,825	
BF_RNAi_2	65,759,217	52,168,736	43,241,850	26,384,091	
PB_RNAi_1	88,031,910	77,238,746	63,974,049	38,804,019	
PB_RNAi_2	63,306,509	40,525,699	33,717,868	17,052,489	
					**Mapped 120 to 180 bp**
LZ_Mnase_1_p	78,869,827	70,998,067	69,999,215	53,710,360	22,544,594
LZ_Mnase_1_m	78,869,827	70,777,523	69,799,526		
LZ_Mnase_2_p	87,927,065	78,974,132	77,873,141	59,322,688	22,940,073
LZ_Mnase_2_m	87,927,065	78,716,690	77,635,884		
BF_Mnase_1_p	82,252,471	73,707,098	72,634,329	55,653,352	27,609,945
BF_Mnase_1_m	82,252,471	73,458,714	72,409,907		
BF_Mnase_2_p	82,219,217	73,337,226	72,256,338	55,198,535	28,159,476
BF_Mnase_2_m	82,219,217	73,075,075	72,013,577		
PB_Mnase_1_p	86,813,028	78,175,489	77,077,144	59,308,872	24,642,671
PB_Mnase_1_m	86,813,028	77,927,984	76,850,334		
PB_Mnase_2_p	89,759,708	80,387,040	79,243,915	60,292,132	26,789,272
PB_Mnase_2_m	89,759,708	80,119,912	78,998,804		
